# The efficacy of intrauterine infusion of platelet rich plasma in women undergoing assisted reproduction: a systematic review and meta-analysis

**DOI:** 10.1186/s12884-023-06140-0

**Published:** 2023-12-08

**Authors:** Noran Magdy Shalma, Hazem Mohamed Salamah, Ashraf Alsawareah, Ahmad Shehata Shaarawy, Mohamed Reyad Mohamed, Emery Manirambona, Mohamed Abd-ElGawad

**Affiliations:** 1 Faculty of Medicine, Tanta University, Othman Ibn-Affan Street, Beside Tanta Sporting Club, Tanta, Egypt; 2 Faculty of Medicine, Zagazig University, Zagazig, Egypt; 3 Faculty of Medicine, The Hashemite University, Zarqa, Jordan; 4 Faculty of Medicine, Al-Azhar University, Cairo, Egypt; 5 College of Medicine and Health Sciences, University of Rwanda, Kigali, Rwanda; 6 Faculty of Medicine, Fayoum University, Fayoum, Egypt

**Keywords:** Assisted reproduction, Platelet rich plasma, In vitro fertilization, PRP, IVF, Repeated implantation failure

## Abstract

**Background:**

Platelet-rich plasma (PRP) is an autologous platelet concentration recently used in the reproductive field. Studies had conflicting results regarding its effect on pregnancy outcomes. We aimed to solve the debate on the safety and efficacy of PRP in women undergoing assisted reproduction and assess the influence of covariates on the outcomes of PRP infusion.

**Methods:**

We searched PubMed, Scopus, Cochrane, and Web of Science in May 2023. We included randomized and non-randomized clinical trials as well as cohort studies assessing intrauterine PRP in sub fertile women undergoing assisted reproduction (IVF/ICSI). For the quality assessment, We used the Cochrane Risk of Bias Tool 1, the ROBINS-I tool, and the Newcastle–Ottawa Scale. We pooled the data using RevMan version 5.4.

**Results:**

The data from 23 studies were pooled. PRP had favorable outcomes compared with the control group on clinical pregnancy rate (RR: 1.84, 95% CI 1.62 to 2.09; P < 0.00001), live birth rate (RR: 1.75, 95% CI: 1.24 to 2.47; *P* = 0.001), and miscarriages (RR: 0.51, 95% CI: 0.36 to 0.72; *P* = 0.0002). Women with repeated implantation failure had a significantly improved clinical pregnancy rate (RR: 1.83, 95% CI: 1.49 to 2.24; *P* < 0.00001), live birth rate (RR:1.83, 95% CI: 1.33 to 2.51; *P* = 0.002), and miscarriage rate (RR: 0.46, 95% CI: 0.31 to 068; *P* = 0.0001).

**Conclusion:**

PRP showed promising results in assisted reproductive techniques. Further large and multicenter RCTs are required to compare the doses of PRP while identifying the specific population with the most benefits from PRP.

**Supplementary Information:**

The online version contains supplementary material available at 10.1186/s12884-023-06140-0.

## Background

Although assisted reproduction techniques significantly improved conception rates, the issue of implantation failures remains unsolved. This can be attributed mostly to poor endometrial receptivity and embryo endometrial communication where achieving an implantation necessitates a receptive endometrium, a functional embryo, and a coordinated communication between them [1]. This happens naturally five to seven days after ovulation. The endometrial receptivity then is optimum for embryo implantation [2]. Endometrial receptivity can be affected by many factors including anatomical uterine abnormalities and endometrial thickness among others [3]. Thin endometrium of less than 7 mm is frequently linked to poor conception outcomes such as recurrent implantation failure (RIF) [4]. RIF is defined as the implantation failure of at least three successive in vitro fertilization (IVF) treatments with good quality embryos [5]. It constitutes a major economic and psychologic problem [6]. Thus, it is essential to find an effective treatment that can improve pregnancy outcomes.

Currently, there is no consensus on the optimal approach. In some women, the hormonal therapy might be unsuccessful in increasing the thickness of the endometrium [7]. Moreover, irrespective of endometrial morphometry, the endometrial blood flow was impaired during follicular phase in patients with unexplained implantation failures [8]. New therapeutic options have been suggested to enhance pregnancy rates for women with implantation failures. These interventions include intra-uterine granulocyte colony stimulating factor (G-CSF), intra-uterine human chorionic gonadotropins, and intra-uterine platelet rich plasma (PRP). The network meta-analysis by Jin et al. revealed that among these interventions, PRP was the most effective among women with 2 or more implantation failures [6].

Platelets are small non-nucleated cellular fragments involved in homeostasis derived from megakaryocytes with a short life span [9].They have granules that store various cytokines, and growth factors. At the site of inflammation or injury, platelets are activated and several factors are released including fibroblast growth factor, platelet-derived growth factor, tumor growth factor-β, and vascular endothelial growth factor. [10]. Therefore, administering a platelet concentrate involves infusing a huge quantity of cytokines and chemokines that enhance immunity, healing, and regeneration. [10]. PRP is an autologous platelet concentration in plasma. For the preparation of PRP, blood is drawn from a peripheral vein, kept in the anticoagulant citrate dextrose solution then processed to enhance platelets by separating distinct components of blood [11]. It has recently been identified as an effective therapy in many fields.

The role of PRP in sub fertile women was first investigated by Chang et al. [12]. They found that PRP improved endometrial thickness and pregnancy outcomes. Therefore, several studies investigated the efficacy of PRP. However, they came with conflicting results. Some studies [13, 14] found no difference in the risk of miscarriages while Nazari et al. [15] showed that PRP had significantly reduced miscarriages. Some studies [16–18] demonstrated that PRP infusion had insignificant effect on clinical pregnancy rate while others [15, 19, 20] showed that PRP improved it significantly. Since the studies had inconsistent results, we conducted our systematic review and meta-analysis to investigate the role of intrauterine infusion of PRP on conception outcomes, and solve the ongoing debate. We also aimed to assess the effect of covariates on the outcomes of PRP infusion.

## Methods

We conducted our systematic review and meta-analysis following the Preferred Reporting Items for Systematic Reviews and Meta-Analyses (PRISMA) statement. [21]. We followed the Cochrane handbook guidelines in doing all the steps [22].

### Search strategy

We searched Cochrane, PubMed, Web of Science, and Scopus in June 2022 then updated it in May 2023. We used the following keywords in our search strategy (“platelet rich plasma” OR “platelet gel” OR PRP) AND (“in vitro fertilization” OR “embryo transfer” OR RIF OR “embryo implantation”). The supplementary file contains the search strategy. We searched clinicalTrials.gov manually, and protocols without published results in a peer reviewed journal were excluded.

### Study selection

Two authors in two steps manually screened the retrieved studies. At first we screened the studies according to their title and abstract then we screened the full-text of eligible studies. For any discrepancies, a third author was consulted. We included randomized clinical trials (RCTs), cohort studies, and non-randomized controlled trials comparing intrauterine infusion of platelet-rich plasma with no PRP or placebo in sub fertile women undergoing assisted reproduction (IVF/ICSI).

Abstracts, reviews, editorials, single arm trials, case series, and non-English studies were excluded.

### Quality assessment

For assessment of the included studies, two authors independently evaluated them. For RCTs, we used the Cochrane risk of bias tool 1 (ROB1) [23]. The judgement of the authors is classified as low risk, unclear risk, or high risk of bias. If there was a disagreement, a third author was consulted. We used the ROBINS-I tool [24] for evaluating the quality of non-randomized studies. We used the Newcastle–Ottawa Scale (NOS) [25] for cohort studies.

### Data extraction and study outcomes

The authors performed the data extraction in prepared formatted excel sheets. The characteristics of the studies included: inclusion and exclusion criteria, study ID, center (country), intervention and control arms, study design, and reported outcomes.

The baseline data included the age, etiology and duration of infertility, body mass index, type of infertility, number of embryos transferred, previous implantation failure, and endometrial thickness.

The primary outcomes were clinical pregnancy, live birth, and miscarriages.

The secondary outcomes were implantation rate, chemical pregnancy, endometrial thickness, ectopic pregnancies, multiple pregnancies, and ongoing pregnancies. Methods indicated in the Cochrane manual were used to deal with any incomplete or incompatible data. [22].

### Statistical analysis

For endometrial thickness, mean difference (MD) and its 95% confidence interval (CI) were calculated, while the risk ratios (RR) with 95% CI were calculated for dichotomous variables. We used a fixed-effect model if there is no heterogeneity (P > 0.05); otherwise, a random-effect model was used. We assessed the statistical heterogeneity using the *I* ^2^ statistic where p- value of less than 0.05, *I* ^2^ more than 60% indicated heterogeneity_._ We conducted sensitivity analysis through exclusion of the study with the highest heterogeneity. We calculated miscarriages, multiple pregnancy, and ectopic pregnancies per the number of clinical pregnancies. Implantation rate was determined as the number of gestational sacs per the overall number of transferred embryos.

We performed the meta-analysis using Revman software 5.4. For the assessment of publication bias, we visually inspected the symmetry of funnel plot. We performed subgroup analysis for women with thin endometrium less than (7 mm), and RIF with 3 or more implantation failures. We performed a meta-regression using open meta-analyst to investigate the influence of age, BMI, duration of infertility, endometrial thickness, number of previous cycles, and number of embryos transferred on clinical pregnancy, chemical pregnancy, and miscarriages.

## Results

### Summary of literature search

**Fig. 1:**
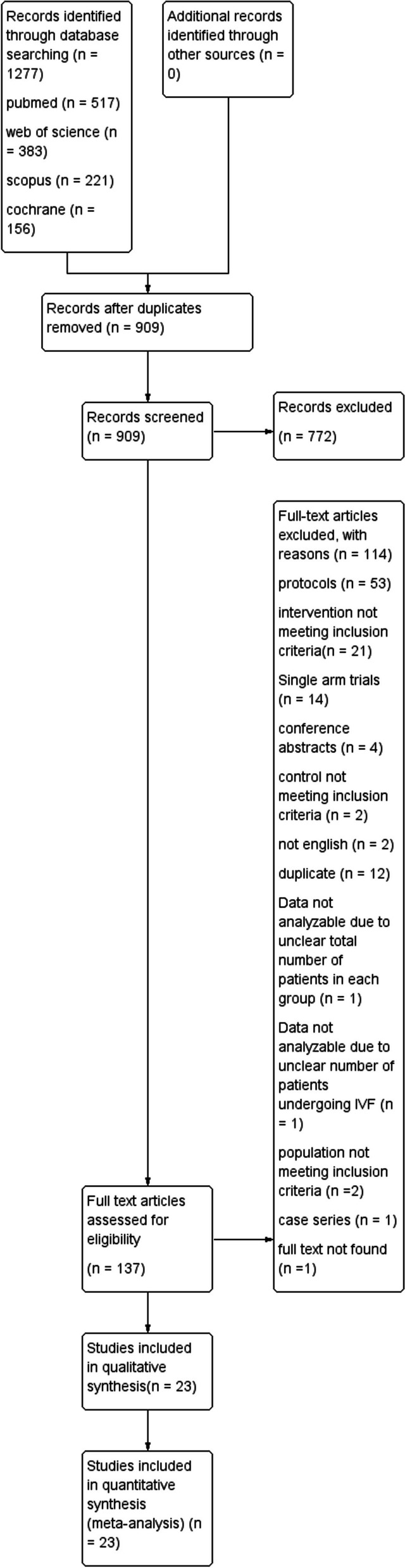
Shows the PRISMA flow chart, which summarizes the literature search, screening, and the number of included studies

Our literature search strategy retrieved 1227 publications; of which 318 were duplicated and removed. Following title and abstract screening, We screened the full text of 137 studies. Of which, 23 were included [13–20, 26–40]. The flowchart demonstrating the studies selection process is presented in Fig. 1.

### Study characteristics

Table 1 demonstrates a summary of the characteristics of the included studies. Studies were carried out between 2014 and 2021, of which 13 studies took place in Iran, four studies in China and a single study from every country of these (Bahrain, Egypt, India, Russia, Turkey, Saudi Arabia, and USA). We have included 14 RCTs [15–17, 20, 29–38], three non-RCTs [18, 26, 28] and six cohort studies [13, 14, 19, 27, 37, 40].

**Table 1:** Summary of the included studies

Study ID	Study Design, country and time of realization	Inclusion criteria	Intervention group	Control group	Outcomes	Causes of subfertility	The platelet concentration of PRP	Time of PRP infusion	Type of embryo transfer
Eftekhar et al. 2018 [29]	RCT, Iran, between September 2016 and January 2017	The inclusion criteria were women aged 18 to 42 years who were candidates for FET due to a poor endometrial response (endometrium thickness less than 7 mm) to conventional hormone replacement treatment on the 13th day of the cycle in FET cycles	*N* = 33. 0.5–1 cc intrauterine infusion of PRP with HRT	*N* = 33. HRT	Endometrial thickness, chemical, clinical, and ongoing pregnancy rates	Male factors, polycystic ovary syndrome, diminished ovarian reserve, tubal factors,endometrio-sis, mixed, unexplained	4–5 times more than circulating blood	13th day of HRT cycle	Frozen-thawed multiple/single, cleavage state
Nazari et al. 2019 [30]	RCT, Iran, between 2016 and 2017	The inclusion criteria were women age ≤ 38 years and body mass index ≤ 30 kg/m2 with a history of cancelled FET cycle owing to inadequate endometrial thickness (≤ 7 mm) despite standard treatments	*N* = 30. 0.5 ml intrauterine infusion of PRP with HRT	*N* = 30. sham catheter with HRT	Endometrial thickness, clinical pregnancy, and chemical pregnancy	Male factor, diminished ovarian reserve, tubal factor, anovulation and mixed	4–5 times more than circulating blood	On day 11–12 of the menstrual cycle	Frozen-thawed embryo transfer cleavage stage
Nazari et al. 2020 [31]	RCT, Iran, between 2016 and 2017	The inclusion criteria were age below 40 years and b BMI below 30 kg/m2 who failed to conceive after 3 or more embryo transfers with high-quality embryos and candidates for FET	*N* = 49. 0.5 ml intrauterine infusion of PRP with HRT	*N* = 48. HRT	Chemical pregnancy, and clinical pregnancy	Male factor, diminished ovarian reserve, tubal factor, anovulation and mixed	4–5 times more than circulating blood	48 h before Embryo transfer	Frozen thawed embryo transfer, blastocyst
Allahveisi et al. 2020 [17]	RCT, Iran, from 2018 to 2019	The inclusion criteria were infertile women with a history of unsuccessful implantation referred to Besat Hospital's Infertility Center in Sanandaj	*N* = 25. 0.5 ml intrauterine infusion of PRP	*N* = 25. 0.5 mL intrauterine infusion of Ringer serum	Implantation rate, clinical pregnancy rate, live birth	Male factor	Range from 411*10^3 to 1067*10^3/μL	48 h before Embryo transfer	Frozen embryo transfer
Rageh et al. 2020 [33]	RCT, Bahrain, from July 2018 to March 2019	Women aged under 40 with a BMI of less than 30 kg/m2 who failed to conceive following three or more ET with high-quality embryos	*N* = 75. 0.5- 1 ml intrauterine infusion of PRP	*N* = 75. No PRP	Chemical pregnancy	Male factor, tubal factor, polycystic ovary syndrome, unexplained	4–5 times more than circulating blood	48 h before Embryo transfer	Multiple embryo transfer, blastocyst
Zamaniyan et al. 2021 [20]	RCT, Iran, from February 2016 to January 2019	Women aged 20–40 years with a BMI of less than 30 kg/m2 and normal hysterosalpingography who were unable to become pregnant following three or more high-quality embryo transfers	*N* = 60. 0.5 ml intrauterine infusion of PRP	*N* = 60. No PRP	Implantation rate, clinical pregnancy rate, chemical pregnancy	Male factor, tubal factor, Polycystic ovary syndrome, unexplained infertility, multiple factors	4–7 times more than circulating blood	48 h before Embryo transfer	Frozen embryo transfer, single or multiple, blastocyst
Zargar et al. 2021 [35]	RCT, Iran	Infertile women under the age of 41 who have had at least two IVF failures	*N* = 40. 1.5 ml intrauterine infusion of PRP	*N* = 40. No PRP	Implantation rate, live birth rate, miscarriages, pregnancy rate	Male factor, female factor, both	NR	48 h before Embryo transfer	Fresh or frozen embryo transfer, single or multiple
Ershadi et al. 2022 [16]	RCT, Iran, 2019	Infertile women visiting this hospital's infertility clinic under the age of 40 and having a history of two to three IVF failures	*N* = 45. 0.5 ml intrauterine infusion of PRP with HRT	*N* = 45. HRT	The rate of implantation, chemical and clinical pregnancies, miscarriage	NR	4–5 times more than circulating blood	48 h before Embryo transfer	Frozen embryo transfer, multiple/single
Safdarian et al. 2022 [34]	RCT, Iran, from October 2017 to April2020	Women between the ages of 20 and 40 who are infertile.and were eligible for FET after failing to conceive following three or more ET using high-quality embryos and having at least one frozen good-quality blastocyst-stage embryo	*N* = 60. 0.5 ml intrauterine infusion of PRP	*N* = 60. No PRP	Implantation rate, live birth, clinical pregnancy, multiple pregnancy, miscarriage	Male factor, female factor, mixed	4–5 times more than circulating blood	48 h before Embryo transfer	Frozen embryo transfer. Single or multiple, blastocyst
Nazari et al. 2022 a [15]	RCT, Iran, between 2018 and 2020	Women who had a history of failure to achieve pregnancy following three or more embryo transfers with high-quality embryos. age between 18 and 38, a BMI of 30 kg/m2, and a serum FSH level of 10 mIU/ml on day 2 or 3 of the menstrual cycle	*N* = 196. 0.5 ml intrauterine infusion of PRP with standard treatment	*N* = 197. standard treatment	The rates of chemical and clinical pregnancy	NR	4–5 times more than circulating blood	48 h before Embryo transfer	Frozen embryo Transfer, blastocyst transfer, Multiple
Nazari et al. 2022 b [32]	RCT, Iran, from December 2019 to August 2020	Women aged below 40 years with two or more pregnancy losses before 20 weeks of gestation who were candidates for ICSI,and had BMI of 20–30 kg/m2	*N* = 20. 0.5 ml intrauterine infusion of PRP with standard treatment	*N* = 20. standard treatment	Ongoing pregnancy, live birth rate, clinical pregnancy, chemical pregnancy, miscarriage rate	Male factor, poly cystic ovary and unexplained	4–5 times more than circulating blood	48 h before Embryo transfer	Fresh embryo transfer, one or two blastocyst embryos
Dzhincharadze et al. 2021 [28]	Non-RCT, Russia	Women aged 20–42 years with body mass index: 18–30 kg/m2,with regular menstrual cycle,normal uterine cavity confirmed by hysteroscopy, history of cancelled embryo transfer due to thin endometrium, infertility due to tubal and/or male factor and/or external genital endometriosis; idiopathic infertility, at least 3 vitrified blastocysts with excellent quality; good quality and/or average quality,	*N* = 37. 5–7 ml intrauterine infusion of PRP with cyclic hormone therapy	*N* = 17. cyclic hormone therapy	Endometrial thickness, clinical pregnancy	Male factors, poor ovarian reserve, tubal factors, combined and other	4–5 times more than circulating blood	8–9, 10–11, and 12–13 days of the menstrual cycle	Frozen-thawed embryo transfer
Tehraninejad et al. 2021 [18]	Non-RCT, Iran, from 2016 to 2018	RIF patients with endometrial thickness more than or equal to 7 mm	*N* = 42. 1 ml intrauterine infusion of PRP	*N* = 20. No PRP	Ongoing pregnancy rate, clinical pregnancy rate, chemical pregnancy	Male factor, female factor, unexplained	4–5 times more than circulating blood	48 h before Embryo transfer	Frozen embryo transfer, multiple, blastocyst
Abou-El-Naga et al. 2022 [26]	Non-RCT, Egypt, from August 2020 to June	Women a history of RIF who had a BMI of 30 kg/m2, aged 18–40 years, and submitted to fresh embryo transfers with good embryos	*N* = 20. 0.5 ml intrauterine infusion of PRP	*N* = 43. No PRP	Endometrial expansion, chemical and clinical pregnancies	NR	1036.7 ± 136.2 (Mean ± SD)	48 h before Embryo transfer	Fresh embryo transfer
Chang et al. 2019 [27]	Prospective cohort, China, from July 2015 to July 2016	Patients with thin endometrium going to receive FET and had at least 2 frozen good-quality blastocyst-stage embryos, who were younger than 40 years with a basal serum FSH < 10 IU/L. with a history of cancelled ET due to thin endometrium (< 7 mm) in HRT cycles, they had no history of hematological disorders, no intrauterine adhesion in the diagnostic hysteroscopy, no submucosal uterine myoma or endometrial polyps	*N* = 34. 0.5- 1 ml intrauterine infusion of PRP	*N *= 30. No PRP	Endometrial thickness, implantation rate, and clinical pregnancy rate	Polycystic ovary syndrome, pelvic or tubal factors, endometriosis or adenomyosis and other causes	889.42 ± 64.41 × 10^3/μL (Mean ± SD)	NR	Frozen embryo transfer. Blastocyst-stage embryo
Coksuer et al. 2019 [19]	Retrospective cohort, Turkey, between January 2014 and January 2017	Patients with body mass index between 18 and 28, aged between 21 and 39 with history of RIF, normal hysteroscopy results, normal karyotype of both couple, a regular menstrual cycle, evidence of ovulation,, normal tubal patency, normal sperm parameters, with no systemic immunologic, endocrine disease or thrombophilia, having three blastocyst FET and could not achieve optimal endometrium lining which was < 7 mm despite appropriate estradiol valerate therapy	*N* = 34. 1 ml intrauterine infusion of PRP	*N* = 36. No PRP	Clinical pregnancy, live birth, endometrial thickness, chemical pregnancy, spontaneous abortion, ectopic pregnancy	NR	992.45 ± 212.85 10^3 cell/mL. (Mean ± SD)	48 h before Embryo transfer	Frozen embryo transfer
Noushin et al. 2021 [13]	Observational prospective cohort, India, from March 2019 to May 2020	Women under the age of 40 with a history of RIF undergoing frozen embryo transfer	*N* = 109. 1 ml intrauterine infusion of PRP	*N* = 154. No PRP	Ongoing pregnancy, live birth rate, clinical pregnancy, chemical pregnancy, miscarriage rate	NR	1,254,770 ± 123,555 (Mean ± SD)	3–7 days before Embryo transfer	Frozen embryo transfer, multiple, cleavage-/ blastocyst-stage
Xu et al. 2022 [14]	Retrospective cohort, China, from October 2019 to January 2021	Patients aged 23 to 40 with good-quality embryos who had three or more successive unsuccessful embryo implantations	*N* = 138. 1 ml intrauterine infusion of PRP	*N* = 150. No PRP	Implantation rate, live birth rate, clinical pregnancy rate, miscarriage rate	Male factor, tubal factor, Polycystic ovary syndrome, others	513.45 ± 322.18 × 10^9/L (Mean ± SD)	48 h before Embryo transfer	Frozen embryo transfer. Single or multiple, blastocyst cleavage stage
Abduljabbar et al. 2022 [36]	RCT, Saudi Arabia, from September 2020, to May 1, 2021	Subjects undergoing IVF/ICSI-frozen embryo transfer (FET) with repeated failures, age between 18 and 44 years, type of infertility eligible for IVF/ICSI, and endometrial thicknesses between 0.4 and 0.7 cm	*N* = 35 0.5 mL of PRP was infused into the uterine cavity	*N* = 35 No PRP	endometrial thickness, clinical pregnancy	Male factor,Unexplained, Ovulatory factor, Endometriosis, Tubal factor	NA	after oocyte pickup	cleaveage, and blastocyst
Baybordi et al. 2022 [38]	RCT, Iran between May 2017 to December 2019	women of childbearing age with a history of RIF during ART treatments	*N* = 48 0.5–1 ml of PRP	*N* = 46 No PRP	chemical, clinical pregnancy, ectopic pregnancy, abortion, live birth	NA	NA	48 h before embryo transfer	blastocyst
Yuan et al. 2022 [40]	retrospective cohort, China from January 2019 to December 2021	patients who had 3 or more embryo transfers with at least 4 high-quality embryos (All patients received fresh embryo transfer), but failed to achieve clinical pregnancy, patients with an age of 25 to 40 years old, patients without endometrial mass and the thickness of endometrium was 7–14 mm, and patients with negative blocking antibody in peripheral blood	*N* = 34 about 1.5 mL	*N* = 30 No PRP	The FSH, LH, and E2. Uterine artery pulsation index (PI) and uterine artery resistance index, embryo implantation, clinical pregnancy rate	tubal, ovarian, others	approximately 4 to 5 times the concentration of circulating blood	NA	fresh embryo transfer
Ban et al. 2023 [37]	A Retrospective Cohort Study, China, from January 2019 to December 2021	failure of clinical pregnancy after 3 ET cycles with at least 4 good-quality cleavage-/blastocyst-stage embryos; women with RIF aged < 40 years undergoing FET; endometrium thickness 8 mm; tubal factor infertility	*N* = 64 1 mL of autologous LP-PRP in the syringe connected to the ET catheter was infused into the uterine cavity	*N* = 54 No PRP	serum beta hCG clinical pregnancy rate (CPR), live birth rate (LBR), and miscarriage rate (MR)	tubal, ovarian, unexplained,	NA	two days before ET	FET, blastocyst- or cleavage-
Pourkaveh et al. 2022 [39]	RCT, Iran between March and December of 2018	RIF patient who underwent FET aged below 40 years and (BMI) below 30 kg/m2	*N* = 11 0.5 ml of PRP was infused into the uterine cavity with the ET Catheter	*N* = 9 No PRP	Leukemia Inhibitory Factor (LIF), clinical pregnancy	NA	platelets at about 4–5 times higher in concentration	48 h before ET	FET

*RCT* Randomized controlled trial, *FET* Frozen embryo transfer, *ET* Embryo transfer, *BMI* Body mass index, *HRT* Hormone replacement therapy, *RIF* Repeated implantation failure, *IVF* In vitro fertilization, *FSH* Follicular stimulating hormone, *ICSI* Intracytoplasmic sperm injection, *NR* Not reported, *PRP* Platelet rich plasma

All trials compared PRP versus placebo or no PRP. The sample size varied from 20 to 438 women. The mean age of the participants was between 29–37. The infusion of PRP that was administered varied by dose and time, 11 studies [15–17, 20, 26, 30–32, 34, 36, 39] administered the PRP with dose = 0.5 mL, four studies [27, 29, 33, 38] with dose 0.5 to 1.0 mL, five studies [13, 14, 18, 19, 37] with dose = 1.0 mL and three studies [28, 35, 40] with dose ≥ 1.5 mL.

Regarding time, most studies administered the PRP 48 h before embryo transfer (15 studies) [14–20, 26, 31–35, 37–39], while three studies [28–30] gave the PRP between 8 to 13 day of menstrual cycle and a single study infused the PRP three days before embryo transfer [13]. A study gave it after oocyte pick up [36], and a study didn’t provide information [40]. The baseline characteristics are in Table 2.

**Table 2:** baseline characteristics of the included studies

Study ID	study groups	age (years) mean(SD)	BMI Mean ( SD)	Basal AMH (ng/ml) Mean ( SD)	Basal LH (IU/l) Mean ( SD)	Basal FSH (IU/ml) Mean ( SD)	Duration of infertility in years Mean ( SD)	Primary infertility Number, percentage	Secondary infertility Number, percentage
Eftekhar et al. 2018 [29]	PRP	31.98 (2.26)	NR	NR	NR	NR	NR	29, 72.5%	11, 27.5%
	control	32.4 (2.63)	NR	NR	NR	NR	NR	31, 72.1%	12, 27.9%
Nazari et al. 2019 [30]	PRP	33.95 (2.76)	24.3 (2.24)	NR	NR	NR	NR	NR	NR
	control	32.33 (4.79)	25.46 (2.68)	NR	NR	NR	NR	NR	NR
Nazari et al. 2020 [31]	PRP	35.37 (3.49)	25.61 (3.13)	NR	NR	NR	NR	NR	NR
	control	34.95 (4.23)	25.46 (2.68)	NR	NR	NR	NR	NR	NR
Allahveisi et al. 2020 [17]	PRP	33 (0.9)	25.96 (0.54)	3.91 (0.7)	4.78 (0.43)	6.42 (0.49)	2.68 (0.12)	19,76%	6, 25%
	control	33.8 (0.54)	25.76 (0.47)	6.87 (1.4)	6.01 (1.1)	6.16 (0.6)	2.9 (0.14)	19, 76%	6, 25%
Rageh et al. 2020 [33]	PRP	29.3(3.5)	26.7 (1.1)	1.81 (0.96)	NR	NR	6.6 (3.7)	NR	NR
	control	29.9 (3.9)	26.6 (1.08)	1.577 (0.84)	NR	NR	6.2 (4.4)	NR	NR
Zamaniyan et al. 2021 [20]	PRP	33.88 (6.32)	26.49 (4.53)	NR	NR	NR	6.12 (4.51)	34, 61.8%	18,32.7%
	control	33.13 (5)	25.03 (3.66)	NR	NR	NR	6.17 (3.5)	36, 83.7%	11.60%
Zargar et al. 2021 [35]	PRP	34.15 (5.14)	NR	NR	NR	NR	7.5 (4.73)	34,85%	6,15%
	control	32.82 (5.18)	NR	NR	NR	NR	6.95 (3.04)	32, 80%	8, 20%
Ershadi et al. 2022 [16]	PRP	31.3 (4.3)	26.5 (3.2)	NR	NR	NR	NR	NR	NR
	control	31.2 (4.8)	27.7 (3)	NR	NR	NR	NR	NR	NR
Safdarian et al. 2022 [34]	PRP	33.4 (4.9)	24.85 (2.84)	2.51 (1.22)	NR	NR	NR	NR	NR
	control	34 (3.73)	25.24 (2.71)	2.64 (2.99)	NR	NR	NR	NR	NR
Nazari et al. 2022 a [15]	PRP	34.11 (3.75)	24.73 (3.53)	4.15 (2.23)	4.14 (0.3)	0.0064 (0.0032)	4.3 (2.3)	155,79.1%	41, 20.9%
	control	33.61 (4.06)	25.19 (3.01)	3.94 (2.75)	3.87 (0.8)	0.0067 (0.0012)	4.6 (1.4)	160, 81.2%	37, 18.8%
Nazari et al. 2022 b [32]	PRP	35.7 (5.1)	26.4 (3.43)	2.93 (1.91)	NR	5.11 (2.68)	5.2 (3.6)	10, 50%	10, 50%
	control	34.75 (4.57)	26.6 (4.23)	2.1 (1.67)	NR	4.28 (2.92)	3.65 (2.15)	5,25%	15, 75%
Dzhincharadze et al. 2021 [28]	PRP	36 (6)	23.13 (3.35)	NR	5.1 (3.5)	0.0079 (0.0063)	5.22 (3.8)	NR	NR
	control	36.8 (6.8)	23.66 (3.5)	NR	4.53 (2.95)	0.00842 (0.00799)	6.74 (5.65)	NR	NR
Tehraninejad et al. 2021 [18]	PRP	32.9 (3)	26.2 (2.8)	2.4 (3.7)	NR	0.0064 (0.0022)	8.9 (6.2)	NR	NR
	control	33.5 (2.5)	26.3 (3.3)	2 (2.7)	NR	0.0063 (0.0024)	11 (7)	NR	NR
Abou-El-Naga et al. 2022 [26]	PRP	31.9(2.5)	27.2 (1.9)	NR	NR	NR	4.6 (1.7)	7,35%	13,65%
	control	26.4 (3.8)	27.6 (2)	NR	NR	NR	5.1 (1.3)	7,35%	13, 65%
chang et al. 2019 [27]	PRP	34.77 (0.75)	22.42 (0.42)	NR	4.8 (1.19)	0.00591 (0.00177)	3.57 (1.82)	NR	NR
	control	32.64 (1.7)	22.39 (0.8)	NR	4.31 (1.32)	0.00636 (0.00184)	3.71 (1.66)	NR	NR
Coksuer et al. 2019 [19]	PRP	29.41 (4.54)	26.35 (4.41)	NR	7.2 (0.93)	0.0073 (0.001225)	7 (3)	NR	NR
	control	28.89 (3.91)	26.78 (3.79)	NR	6.5 (0.38)	0.0069 (0.00155)	8 (2.75)	NR	NR
Noushin et al. 2021 [13]	PRP	32.28 (4.84)	26.28 (0.89)	3.87 (3.03)	NR	NR	5.99 (1.78)	NR	NR
	control	33.01 (4.27)	26.32 (0.93)	4.14 (2.62)	NR	NR	5.33 (1.77)	NR	NR
Xu et al. 2022 [14]	PRP	34.92 (4.8)	24.08 (3.65)	3.38 (2.45)	NR	6.69 (1.5)	4.35 (3.29)	75, 54%	63, 46%
	control	34.93 (4.87)	24.81 (3.9)	2.4 (3.03)	NR	6.45 (1.47)	4.28 (3.74)	78, 52%	72, 48%
Abduljabbar et al. 2022 [36]	PRP	35.91 (4.49)	NR	NR	NR	NR	NR	16, 45.7%	19, 54%
	control	34.63 (4.26)	NR	NR	NR	NR	NR	14, 40%	21, 60%
Baybordi et al. 2022 [38]	PRP	37.33 (6.439)	26.64 (3.302)	NR	NR	NR	12 (6.16)	36, 75%	12, 25%
	control	32.41 (5.651)	26.86 (3.63)	NR	NR	NR	7 (4.71)	39, 85%	7, 15%
Yuan et al. 2022 [40]	PRP	≥ 35 year 19 < 35 year 11	NR	NR	5.78 (2.49)	0.00845 (0.00204)	≥ 4 year 17 < 4 year 13	NR	NR
	control	≥ 35 year 26 < 35 year 8	NR	NR	5.68 (2.01)	0.00851 (0.00218)	≥ 4 year 19 < 4 year 15	NR	NR
Ban et al. 2023 [37]	PRP	32.04 (4.36)	20.69 (4.36)	4.72 (3.84)	6.32 (5.38)	0.00664 (0.00321)	3 (1.52)	NR	NR
	control	32.22 (4.31)	22.52 (2.99)	4.82 (3.56)	5.15 (2.7)	0.00578 (0.00227)	3.5 (2.28)	NR	NR
Pourkaveh et al. 2022 [39]	PRP	35.18 (2.09)	23.09 (1.3)	NR	NR	NR	5.91 (0.94)	NR	NR
	control	34.67 (2)	23.11 (2.09)	NR	NR	NR	6.11 (1.05)	NR	NR

*BMI* Body mass index, *FSH* Follicular stimulating hormone, *LH* Luteinizing hormone, *AMH* Anti-mullerian hormone, *PRP* Platelet-rich plasma, *NR* Not reported

### Quality assessment

The included RCTs were appraised using the Cochrane ROB 1. As for the random sequence generation domain, all the studies were considered low risk except for one study [17] that was at high risk and two studies [33, 39] whose risk was unclear. Regarding the allocation concealment domain, there was inadequate information in most of the studies to permit judgment of low or high risk. However, three studies [20, 35, 38] were considered to be of low risk, and one [17] was judged as high risk.

All studies were at low risk for performance and detection biases.

Regarding missing data, four trials were a source of a high risk of attrition bias, two of them [15, 32] had a significant percentage of loss to follow-up and the other two [20, 39] had unequal distribution to the loss of follow up, the remaining trials had a low risk of attrition bias.

Regarding reporting bias, all studies had low risk except for three trials [17, 31, 32] that did not report one of the primary outcomes and one study that did not provide sufficient information to judge [39].

Most studies were free from any other source of bias, except for five studies, which carried a high risk of bias. Pourkaveh et al. [39] had small study size. Allahveisi et al. [17], had small study size and vagueness regarding the causes of RIF. In Rageh et al. [33], the provided NCT was not found. Safdarian et al. [34] reported that the live birth rate was more than clinical pregnancy rate while Zargar et al. [35] had a wide range of age in its participants.

**Fig. 2:**
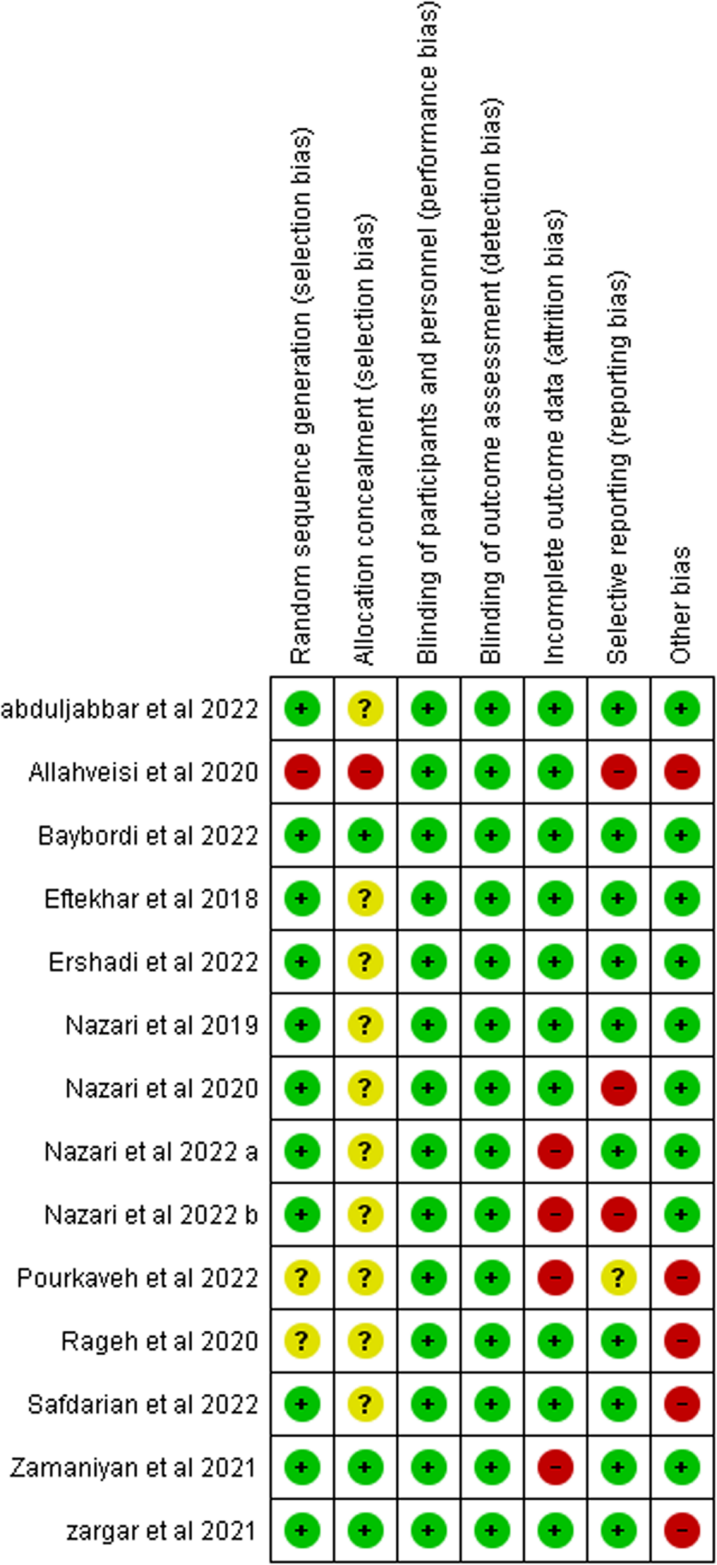
Risk of bias summary. It shows a summary of the risk of bias for each included study

**Fig. 3:**
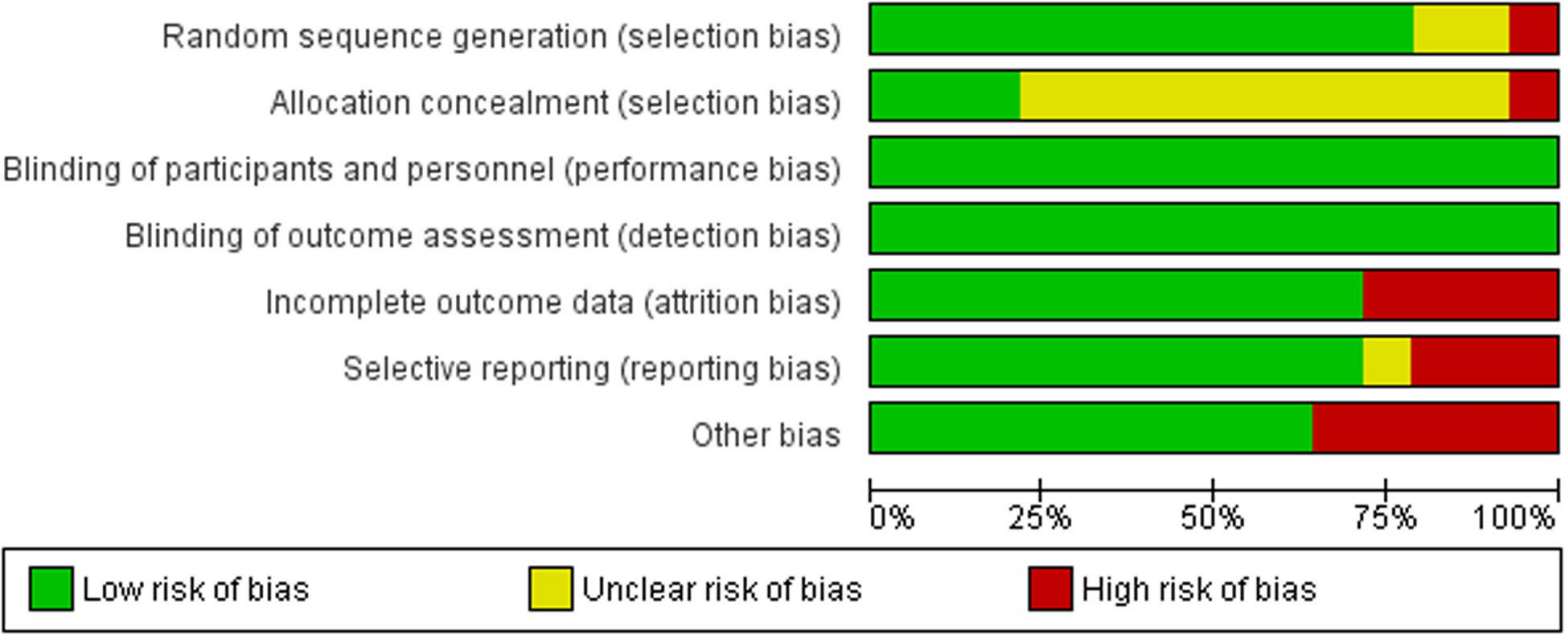
Risk of bias graph for included studies

The summary of the quality assessment for the included 14 RCTs is shown in Figs. 2 and  3.

Regarding the assessment of the three included non-randomized trials, and depending on the three domains of ROBINS-I, Abou-El-Naga et al. [26] were judged to have low risk, but both Dzhincharadze et al. [28] and Tehraninejad et al. [18] were judged to carry moderate to high risk of bias. See the Supplementary Table 1 for the details regarding the scoring of the non-RCTs.

Regarding the three domains of NOS (Selection, Comparability and Outcome), all included studies had good quality except for two studies had fair quality [38, 39]. See Supplementary Table 2 for the details regarding the scoring of the cohort studies according to NOS.

To investigate publication bias, we conducted funnel plots. Supplementary Figures 1 and 2 display the funnel plots for the clinical pregnancy rate and the chemical pregnancy rate in the entire population, respectively, the funnel plot exhibits asymmetry at the bottom, suggesting that studies with unfavorable results and smaller sample sizes were underrepresented, potentially indicating publication bias. For the miscarriage rate, in Supplementary Fig. 3, the funnel plot displays an asymmetrical appearance, indicating the possibility of publication bias.

### Outcomes

#### All population

##### Clinical pregnancy

**Fig. 4:**
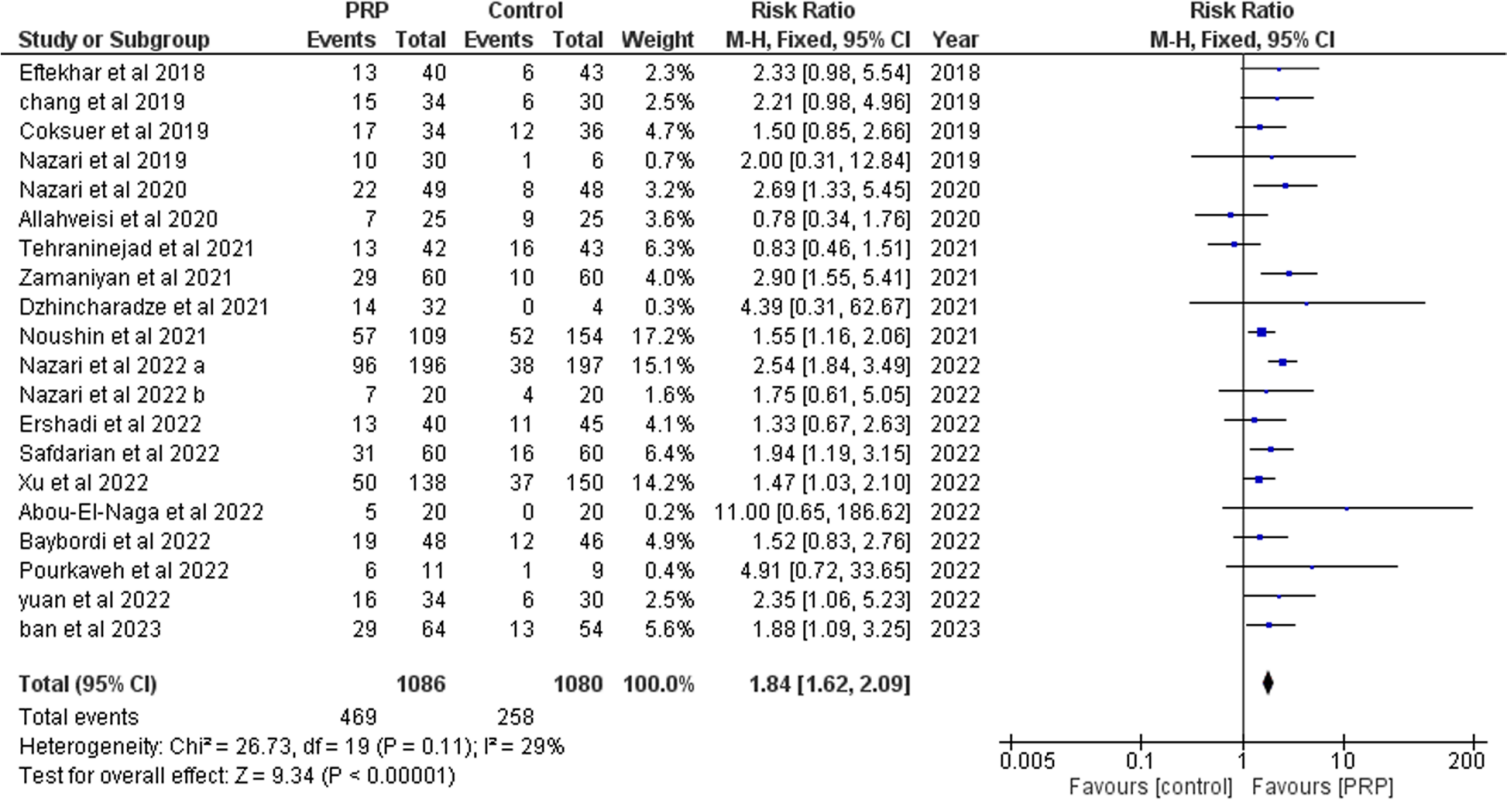
Forrest plot for the effect of PRP-therapy on clinical pregnancy rate. (CI: Confidence Interval, PRP: Platelet Rich Plasma)

Pooling results from 20 studies, [13–20, 26–32, 34, 37–40], including 2166 participants (1086 cases and 1080 controls), showed that clinical pregnancy was significantly higher in the PRP group (RR: 1.84, 95% CI 1.62 to 2.09; *P* < 0.00001, Fig. 4). There was low heterogeneity between studies (*P* = 0.11; I2 = 29%).

##### Live birth

**Fig. 5:**
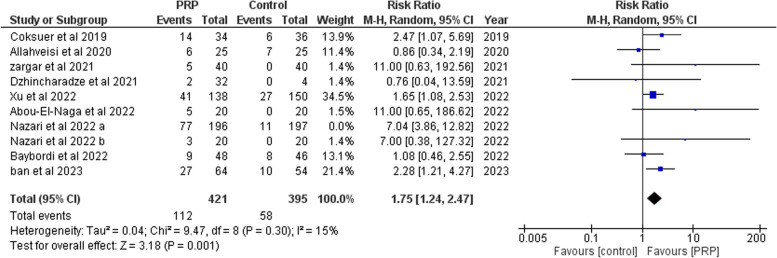
Forrest plot for the effect of PRP-therapy on live birth rate after leave one out. (CI: Confidence Interval, PRP: Platelet Rich Plasma)

A total of ten studies [13, 14, 17, 19, 26, 28, 32, 35, 37, 38] (617 cases and 592 controls) were analyzed. PRP significantly improved live birth (RR: 2.31, 95% CI: 1.33 to 4.02; P = 0.003, Supplementary Fig. 4). There was heterogeneity (*P*= 0.0009, I2= 68%) between studies. However, after leaving Nazari et al 2022 a [15], nine studies [14, 17, 19, 26, 28, 32, 35, 37, 38] (421 cases and 395 controls), live birth was significantly higher in women who received PRP (RR: 1.75, 95% CI: 1.24 to 2.47; *P* = 0.001, Fig 5). There was no heterogeneity (*P*= 0.30, I2= 15%) between studies.

##### Miscarriages

**Fig. 6:**
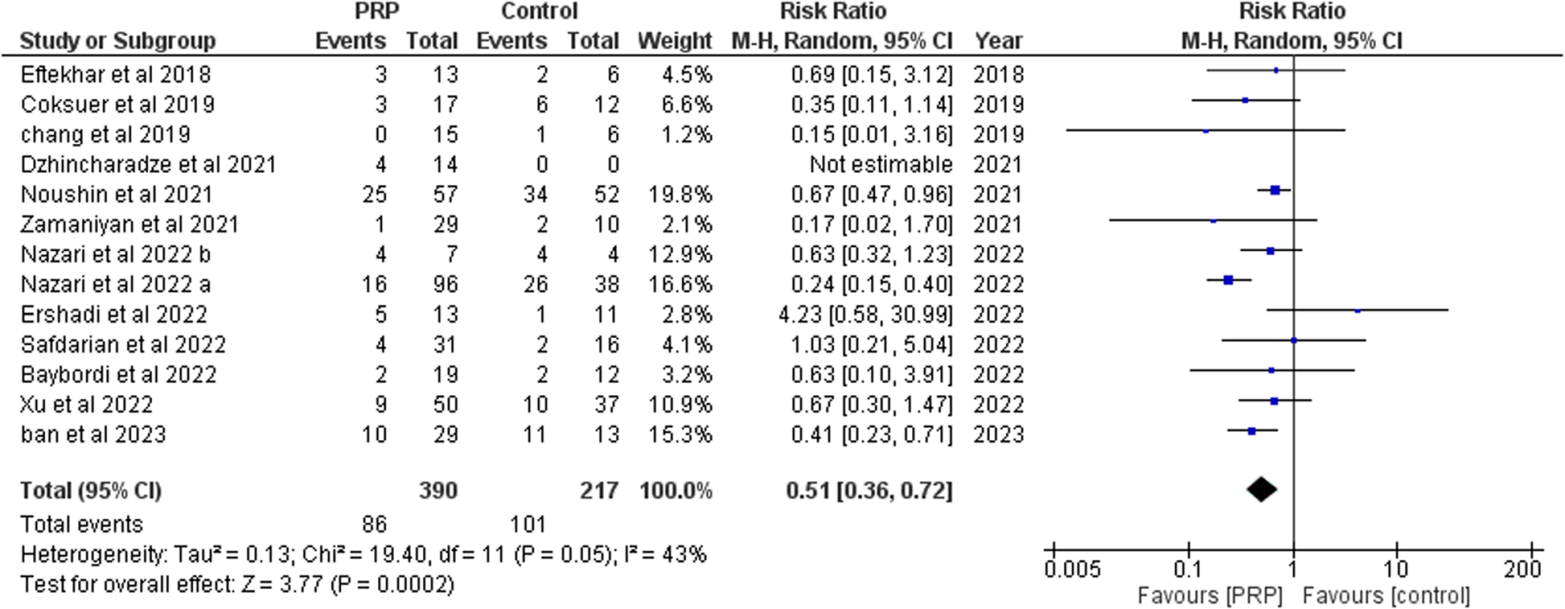
Forrest plot for the effect of PRP-therapy on miscarriages. (CI: Confidence Interval, PRP: Platelet Rich Plasma)

We retrieved 13 studies [13–16, 19, 20, 27–29, 32, 34, 37, 38] with 607 subjects (390 cases and 217 controls). PRP significantly decreased miscarriages compared to the controls (RR: 0.51, 95% CI: 0.36 to 0.72; *P* = 0.0002, Fig. 6). There was homogeneity (*P* = 0.05, I^2^ = 43%) between studies.

##### Endometrial thickness

Changes in endometrial thickness were investigated in six studies [15, 27, 29, 30, 36, 37] (392 cases and 379). A significant difference was observed (MD: 0.82, 95% CI: 0.35 to 1.29; *P* = 0.0007, Fig. 6). There was high heterogeneity (*P*= 0.0001, I^2^= 80%) between studies.

##### Implantation rate

There were seven studies [14, 16, 17, 27, 29, 34, 40]1373 subjects (673 cases and 700 controls). The implantation rate was significantly increased in the PRP arm (RR: 1.63, 95% CI: 1.17 to 2.26; *P*= 0.003, Supplementary Fig. 6). heterogeneities were noticed between studies (*P*= 0.03, I^2^= 57%). After leaving Ershadi et al 2022 [16] six studies, including 1159 subjects (573 cases and 586 controls). PRP significantly increased the implantation rate (RR: 1.81, 95% CI: 1.43 to 2.29; *P*< 0.00001, Supplementary Fig. 7). The studies were homogeneous (*P*= 0.43, I^2^= 0%).

##### Chemical pregnancy

There were 17 studies [13–16, 18–20, 26, 28–31, 33, 34, 36–38] with 2148 participants (1072 cases and 1076 controls). In comparison with the control group, PRP significantly increased chemical pregnancy rate (RR: 1.73, 95% CI: 1.54 to 1.93; *P*< 0.00001, Supplementary Fig. 8). The studies were homogeneous (*P*= 0.45, I^2^= 0%).

##### Ongoing pregnancy

Five studies [18, 20, 29, 34, 38] that included 485 participants (243 cases and 242 controls) were included. PRP significantly increased the ongoing pregnancy rate (RR: 2.13, 95% CI: 1.54 to 2.94; *P*< 0.00001, Supplementary Fig. 9). There was homogeneity (*P*= 0.11, I^2^= 47%) between studies.

##### Ectopic pregnancy

Pooling results from six studies [15, 19, 27, 31, 32, 38], including 256 participants (176 cases and 80 controls), After comparing both groups, we found no major difference in ectopic pregnancy between them. (RR: 0.82, 95% CI 0.27 to 2.45; *P*= 0.72, Supplementary Fig.10). The studies were homogenous (*P*= 0.94, I^2^= 0%).

##### Multiple pregnancy

Four trials [15, 20, 27, 34]were analyzed; they had 241 participants (171 cases and 70 controls). There was insignificant difference in the rate of multiple pregnancy (RR: 1.25, 95% CI 0.57 to 2.76; *P* = 0.58, Supplementary Fig. 11). There was homogeneity between the studies (*P* = 0.95, I^2^ = 0%).

#### Subgroup based on study design (RCT only)

##### Clinical pregnancy

We pooled the results from 11 RCTs [15–17, 20, 29–32, 34, 38, 39], including 1138 participants (579 cases and 559 controls). PRP significantly improved clinical pregnancy rate (RR: 2.12, 95% CI 1.76 to 2.56; *P* < 0.00001, Supplementary Fig. 12). There was homogeneity between studies (*P* = 0.26; I^2^ = 24%).

##### Live birth

Five RCTs [15, 17, 32, 35, 38] (329 cases and 328 controls) were analyzed. No significant difference was found in the live birth rate (RR: 2.72, 95% CI: 0.79 to 9.31; *P* = 0.11, Supplementary Fig. 13). There was heterogeneity (*P* = 0.0002, I^2^ = 82%) between studies. After leaving Nazari et al. 2022 a [15], four RCTs were included [17, 32, 35, 38] (133 cases and 131 controls), there was no significant difference in live birth rate (RR: 1.41, 95% CI: 0.57 to 3,46; *P* = 0.46, Supplementary Fig. 14). The studies were homogeneous (*P* = 0.19, I^2^ = 37%).

##### Miscarriages

We retrieved seven RCTs [15, 16, 20, 29, 32, 34, 38] with 305 subjects (208 cases and 97 controls). No major difference was found in the rate of miscarriage (RR: 0.56, 95% CI: 0.28 to 1.12; *P* = 0.10, Supplementary Fig. 15). There was heterogeneity (*P* = 0.03, I^2^ = 56%) between studies. After leaving one out [15], we included six RCTs [16, 20, 29, 32, 34, 38] with 171 participants (112 cases and 59 controls). PRP had insignificant effect on miscarriage rate (RR: 0.72, 95% CI: 0.41 to 1.27; *P* = 0.26, Supplementary Fig. 16). There was no heterogeneity (*P* = 0.37, I^2^ = 7%) between studies.

#### RIF

##### Clinical pregnancy

Pooling data from 13 studies [13–15, 18–20, 26, 31, 34, 37–40] involving 1772 subjects (865 cases and 907 controls). The clinical pregnancy rate was significantly increased (RR: 1.83, 95% CI: 1.49 to 2.24; *P* < 0.00001, Supplementary Fig. 17). The studies were homogeneous (*P* = 0.05, I^2^ = 43%).

##### Live birth

A total of six studies [14, 15, 19, 26, 37, 38] (500 cases and 503 controls) were included in the analysis. The PRP arm had a significantly higher live birth rate (RR: 2.54, 95% CI: 1.36 to 4.73; *P* = 0.003, Supplementary Fig. 18). There was heterogeneity (*P* = 0.0008, I^2^ = 76%) between studies. After leaving Nazari et al. [15], five studies [14, 19, 26, 37, 38] with 610 subjects (304 cases and 306 controls) were included. PRP significantly increased live birth rate (RR:1.83, 95% CI: 1.33 to 2.51; *P* = 0.002, Supplementary Fig. 19). The studies were homogeneous (*P* = 0.38, I^2^ = 5%).

##### Miscarriages

We retrieved eight studies [13–15, 19, 20, 34, 37, 38] with 518 subjects (328 cases and 190 controls). PRP administration significantly reduced the rate of miscarriage (RR: 0.46, 95% CI: 0.31 to 068; *P* = 0.0001, Supplementary Fig. 20). The studies were homogeneous. (*P* = 0.06, I^2^ = 49%).

#### Thin endometrium

##### Clinical pregnancy

Pooling data from five studies [19, 27–30], including 289 participants (170 cases and 119 controls). PRP infusion significantly increased clinical pregnancy rate (RR: 1.98, 95% CI: 1.32 to 2.97; *P* = 0.001, Supplementary Fig. 21). The studies were homogeneous (*P* = 0.83; I^2^ = 0%).

##### Miscarriages

We retrieved four studies [19, 27–29] with 83 subjects (59 cases and 24 controls). Miscarriage rate was significantly reduced in the PRP arm compared to the controls (RR: 0.39, 95% CI: 0.17 to 0.94; *P* = 0.04, Supplementary Fig. 22). There was homogeneity between studies (*P* = 0.61, I^2^ = 0%).

##### Subgroup analysis based on dose and age

Subgroup analysis based on doses of PRP showed that PRP at a dose of 0.5 ml significantly increased the clinical pregnancy [RR = 2.23, 95% CI (1.82, 2.73), *P* < 0.00001], and the pooled analysis was homogeneous (I^2^ = 29%, *P* = 0.18). PRP was comparable to the control in terms of miscarriage [RR = 0.56, 95% CI (0.23, 1.35), *P* = 0.20].The pooled analysis was heterogeneous (I^2^ = 69%, *P* = 0.01), however, after leaving Nazari 2022a [15], it was homogenous (I^2^ = 46%, *P* = 0.13), with no significant difference between the 2 groups [RR = 0.82, 95% CI (0.29, 2.27), *P* = 0.70] (Supplementary Figs. 24, 25**)**. PRP had no significant effect on live birth [RR = 3.79, 95% CI (0.84, 17.08), *P* = 0.08]. There was heterogeneity in the pooled analysis (I^2^ = 80%, *P* = 0.002). However, after leaving Allahvesi [17], PRP significantly improved live birth[RR = 7.17, 95% CI (4.03, 12.74), *P* < 0.00001].The analysis was homogeneous(I^2^ = 0%, *P* = 0.95) (Supplementary Figs. 23, 26, 27**)**.

PRP of dose 0.5 to 1 ml significantly improved clinical pregnancy [RR = 1.89, 95% CI (1.24, 2.88) *P* = 0.003], but had no significant effect on miscarriages [RR = 0.55, 95% CI (0.19, 1.63), *P* = 0.28] or live birth [RR = 1.08, 95% CI (0.46, 2.55) *P* = 0.86]. The analysis was homogenous for clinical pregnancy, and miscarriage (I^2^ = 0%, *P* = 0.64), (I^2^ = 0%, *P* = 0.66), respectively. PRP at a dose of one ml significantly reduced miscarriage [RR = 0.57, 95% CI (0.44, 0.75), *P* < 0.0001] and increased live birth [RR = 1.91, 95% CI (1.38, 2.65), *P* < 0.0001] and clinical pregnancy [RR = 1.47, 95% CI (1.22, 1.76), *P* < 0.0001] compared to the control. The pooled analysis was homogeneous for all the outcomes (I^2^ = 1%, *P* = 0.39), (I^2^ = 0%, *P* = 0.57), and (I^2^ = 10%, *P* = 0.35), respectively(Supplementary Figs. 23, 24, 26**)**.

PRP dose of 1.5 ml significantly improved clinical pregnancy [RR = 2.35, 95% CI (1.06, 5.23), *P* = 0.04], but had no significant effect on live birth [RR = 11, 95% CI (0.63, 192.56), *P* = 0.10]. PRP dose of 5–7 ml had no significant effect on clinical pregnancy [RR = 4.39, 95% CI (0.31, 62.67), *P* = 0.27] or live birth [RR = 0.76, 95% CI (0.04, 13.59), *P* = 0.85] (Supplementary Fig. 23, 26**)**.

Subgroup analysis showed that PRP in the subgroup population who were < 35 years old significantly reduced miscarriage [RR = 0.50, 95% CI (0.40, 0.63), *P* < 0.00001] and increased live birth [RR = 2.31, 95% CI (1.29, 4.16), *P* = 0.005] and clinical pregnancy [RR = 1.79, 95% CI (1.57, 2.04), *P* < 0.00001] compared to the control. The pooled analysis was homogeneous for clinical pregnancy (I^2^ = 38%, *P* = 0.06).The analysis was heterogeneous for miscarriage (I^2^ = 47%, *P* = 0.04), however, after leaving Nazari et al. 2022a [15]**,** the analysis was homogenous (I^2^ = 4%, *P* = 0.40) without affecting the pooled effect [RR = 0.59, 95% CI (0.44, 0.78), *P* = 0.0002]. There was heterogeneity in the analysis of live birth (I^2^ = 74%, *P* = 0.0004), but after excluding Nazari et al. 2022a [15]**,** the pooled analysis revealed homogeneity (I^2^ = 27%, *P* = 0.22) without affecting the pooled effect [RR = 1.74, 95% CI (1.19, 2.54), *P* = 0.004]. (Supplementary Figs. 28–32**)**.

Regarding age group ≥ 35 years old, PRP significantly increased clinical pregnancy [RR = 2.52, 95% CI (1.41, 4.50), *P* = 0.002], but was comparable in terms of live birth [RR = 2.29, 95% CI (0.24, 21.72), *P* = 0.47], and miscarriages[RR = 0.63, 95% CI (0.32, 1.23), *P* = 0.17]. Pooled analysis was homogeneous for clinical pregnancy (I^2^ = 0%, *P* = 0.72) and live birth (I^2^ = 17%, *P* = 0.27) (Supplementary Figs. 28, 29, 31**)**.

#### Meta regression

For all the population, there was no significant relation between age, BMI, duration of infertility, endometrial thickness, number of previous cycles, and number of embryos transferred on clinical pregnancy, chemical pregnancy, and miscarriages.

For women with RIF, regarding chemical pregnancy, no significant relation was found with age, BMI, and duration of infertility. Regarding clinical pregnancy, we found no significant relation with age, BMI, and number of previous cycles. There was a significant relation between duration of infertility and clinical pregnancy rate (95% CI: 0.0 to 0.047; *P* = 0.049, Supplementary Fig. 33).

For women with implantation failures, regarding chemical pregnancy, we found no significant relation with age, BMI, and duration of infertility. Regarding clinical pregnancy, there was no significant relation with age, BMI, and duration of infertility. There was a significant relation between number of previous cycles and clinical pregnancy rate (95% CI: 0.019 to 0.584; *P* = 0.037, Supplementary Fig. 34).

## Discussion

We investigated the role of intrauterine PRP among sub fertile women undergoing assisted reproduction and the effect of covariates on the outcomes of PRP. Our systematic review included 23 studies with 2,449 patients. There were 1,229 women receiving intrauterine platelet rich plasma and 1,220 women in the control group. Our analysis on all the included women revealed that PRP significantly improved clinical pregnancy, live birth, miscarriages, implantation rate, chemical pregnancy, ongoing pregnancy, and endometrial thickness whereas insignificant on multiple pregnancy, ectopic pregnancy. As for clinical pregnancy, the same findings were found among the analysis of RCTs, RIF, and thin endometrium. For RIF patients, PRP significantly improved live birth but had no significant effect in the analysis of RCTs. There was no statistically significant effect on miscarriages in the analysis of RCTs, whereas significant among women with RIF and those with thin endometrium. We found a statistically significant relation between clinical pregnancy and the duration of infertility among women with RIF, and with the number of previous cycles among women with implantation failure.

The role of PRP was first investigated in the meta-analysis conducted by Maleki-Hajiagha et al. [41]. However, since the meta-analysis was the first to be conducted, some limitations were considered as they didn’t investigate the role of PRP in live birth, their analysis was based on only 7 studies with 625 women. In one them [42], the control group were on systemic G-CSF. In contrast, among our included studies, systemic G-CSF was administered in both groups in Nourshin et al. [13]. Among the overall population, our results were consistent with them regarding chemical pregnancy, clinical pregnancy, and implantation rates, and endometrial thickness but with larger sample size. However, our results came conflicting regarding miscarriages.

Several meta-analyses were carried out afterwards to investigate the role of PRP in the reproductive field. However, each study had some limitations. Liu et al. [43] combined the effect of invasive sub-endometrial and non-invasive intrauterine infusion. li et al. [44] didn’t follow a strict definition for RIF. Maged et-al. [45] included self-controlled trials. The results of the meta-analyses showed that PRP improved clinical pregnancy [43–47], however they had inconsistent results regarding the risk of miscarriages. Regarding RIF patients, our results were conflicting with the meta-analyses conducted by Anitua et al., li et al., and were consistent with deng et al., and liu et al. [43, 44, 46, 47].

The conflict among the previous meta-analyses on the risk of miscarriages among RIF patients can be attributed to many factors including the criteria for defining RIF, and the number of included studies. The inconsistency in results among the studies as concluded by Noushin et al. is attributable to the absence of consensus on the ideal method for preparation of PRP. Most of the studies did not mention the platelet or white blood cells quantification used in the PRP which would highly influence the results [13].

The role of PRP in improving pregnancy outcomes was believed to be related to its effect on endometrial thickness ever since the study conducted by Chang et al. [12], as there was an association between them. However, this is still questionable. Kim et al. [48] found that although PRP had favorable effect on pregnancy outcomes, no association was found between them. Moreover, it has been suggested that endometrial thickness is a poor predictor of clinical pregnancy [1]. In our study, PRP significantly improved clinical pregnancy and endometrial thickness. However, in the meta-regression, we found no significant relation between them.

The precise mechanism behind PRP's positive impact is still unknown. However, it is suggested that this effect is due to its immunological role where providing an anti-inflammatory endometrial environment hinders the rejection of implantation [49, 50]. This is done through the regulation of several inflammatory cytokines including interleukin1, interleukin 8, and interleukin 1-β [41].

Our strengths is that our review is comprehensive with large sample size. We followed PRISMA guidelines. All our included RCTs were considered low risk in performance bias and detection bias. We investigated the role of PRP on the risk of ectopic pregnancy. This outcome wasn’t investigated in the previous meta-analyses. Our included studies come from 9 different countries across different continents, so our results could be generalizable. We didn’t combine the effect of different methods of PRP administration in contrast to the meta-analysis by Liu et al. We followed a strict definition for RIF patients.

Our limitations is that we only considered studies in English. Most of our included RCTs had unclear allocation concealment. Publication bias was observed among the included studies. There was heterogeneity in the analysis as there was heterogeneity among the studies in the methods of preparation of PRP and subsequently the heterogeneity in the concentrations of platelets used for each dose of therapy. Where the same dose of PRP had different concentrations of platelets across different studies.

A standardized protocol is needed for the preparation of PRP in order to investigate the optimum dose for therapy. We recommend that further RCTs should investigate the optimum dose of PRP, and the role of PRP for different causes cause of subfertility.

## Conclusion

PRP improved clinical pregnancy, live birth, and miscarriage rates in women undergoing IVF/ICSI. Further RCTs are needed to investigate the optimum dose of PRP.

### Supplementary Information

**Additional file 1: Supplementary Table 1.** Quality assessment of the nonrandomized trials using ROBINS-I. **Supplementary Table 2.** Methodological quality assessment of the included 4 studies, based on the NOS for assessing the quality of epidemiological studies.  **Supplementary Fig. 1.** Funnel plot of the meta-analysis of published studies for the clinical pregnancy rate. (RR: Relative Risk, SE: Standard Error). **Supplementary Fig. 2.** Funnel plot of the meta-analysis of published studies for the chemical pregnancy rate. (RR: Relative Risk, SE: Standard Error). **Supplementary Fig. 3.** Funnel plot of the meta-analysis of published studies for the miscarriage rate. (RR: Relative Risk, SE: Standard Error). **Supplementary Fig. 4.** Forrest plot for the effect of PRP-therapy on the live birth rate before leave one out. (CI: Confidence Interval, PRP: Platelet Rich Plasma). **Supplementary Fig. 5.** Forrest plot for the effect of PRP-therapy on endometrial thickness change. (CI: Confidence Interval, PRP: Platelet Rich Plasma). **Supplementary Fig. 6.** Forrest plot for the effect of PRP-therapy on implantation rate before leave one out. (CI: Confidence Interval, PRP: Platelet Rich Plasma). **Supplementary Fig. 7.** Forrest plot for the effect of PRP-therapy on implantation rate after leave one out. (CI: Confidence Interval, PRP: Platelet Rich Plasma). **Supplementary Fig. 8.** Forrest plot for the effect of PRP-therapy on chemical pregnancy rate. (CI: Confidence Interval, PRP: Platelet Rich Plasma). **Supplementary Fig. 9.** Forrest plot for the effect of PRP-therapy on ongoing pregnancy rate. (CI: Confidence Interval, PRP: Platelet Rich Plasma). **Supplementary Fig. 10.** Forrest plot for the effect of PRP-therapy on ectopic pregnancy rate. (CI: Confidence Interval, PRP: Platelet Rich Plasma). **Supplementary Fig. 11**. Forrest plot for the effect of PRP-therapy on multiple pregnancy rate. (CI: Confidence Interval, PRP: Platelet Rich Plasma). **Supplementary Fig. 12.** Forrest plot for the effect of PRP-therapy on clinical pregnancy rate in RCT studies. (CI: Confidence Interval, PRP: Platelet Rich Plasma). **Supplementary Fig. 13.** Forrest plot for the effect of PRP-therapy on the live birth rate before leave one out in RCT studies. (CI: Confidence Interval, PRP: Platelet Rich Plasma). **Supplementary Fig. 14.** Forrest plot for the effect of PRP-therapy on live birth rate after leave one out in RCT studies. (CI: Confidence Interval, PRP: Platelet Rich Plasma). **Supplementary Fig. 15.** Forrest plot for the effect of PRP-therapy on miscarriage rate before leave one out in RCT studies. (CI: Confidence Interval, PRP: Platelet Rich Plasma). **Supplementary Fig. 16.** Forrest plot for the effect of PRP-therapy on miscarriage rate after leave one out in RCT studies. (CI: Confidence Interval, PRP: Platelet Rich Plasma). **Supplementary Fig. 17.** Forrest plot for the effect of PRP-therapy on clinical pregnancy rate in patients with RIF (CI: Confidence Interval, PRP: Platelet Rich Plasma). **Supplementary Fig.18.** Forrest plot for the effect of PRP-therapy on the live birth rate before leave one out in patients with RIF. (CI: Confidence Interval, PRP: Platelet Rich Plasma). **Supplementary Fig. 19.** Forrest plot for the effect of PRP-therapy on the live birth rate after leave one out in patients with RIF. (CI: Confidence Interval, PRP: Platelet Rich Plasma). **Supplementary Fig. 20.** Forrest plot for the effect of PRP-therapy on miscarriage rate in patients with RIF. (CI: Confidence Interval, PRP: Platelet Rich Plasma). **Supplementary Fig. 21.** Forrest plot for the effect of PRP-therapy on clinical pregnancy rate one in patients with thin endometrium. (CI: Confidence Interval, PRP: Platelet Rich Plasma). **Supplementary Fig. 22.** Forrest plot for the effect of PRP-therapy on miscarriage rate in patients with thin endometrium. (CI: Confidence Interval, PRP: Platelet Rich Plasma). **Supplementary Fig. 23.** Forrest plot for the effect of PRP-therapy on clinical pregnancy rate. (CI: Confidence Interval, PRP: Platelet Rich Plasma). **Supplementary Fig. 24.** Forrest plot for the effect of PRP-therapy on miscarriages. (CI: Confidence Interval, PRP: Platelet Rich Plasma). **Supplementary Fig. 25.** Forrest plot for the effect of PRP-therapy on miscarriages after leaving Nazari 2022a. (CI: Confidence Interval, PRP: Platelet Rich Plasma). **Supplementary Fig. 26.** Forrest plot for the effect of PRP-therapy on live birth. (CI: Confidence Interval, PRP: Platelet Rich Plasma). **Supplementary Fig. 27.** Forrest plot for the effect of PRP-therapy on live birth after allahveisi. (CI: Confidence Interval, PRP: Platelet Rich Plasma). **Supplementary Fig. 28.** Forrest plot for the effect of PRP-therapy on clinical pregnancy. (CI: Confidence Interval, PRP: Platelet Rich Plasma). **Supplementary Fig. 29.** Forrest plot for the effect of PRP-therapy on miscarriage. (CI: Confidence Interval, PRP: Platelet Rich Plasma). **Supplementary Fig. 30.** Forrest plot for the effect of PRP-therapy on miscarriage after leaving Nazari 2022a. (CI: Confidence Interval, PRP: Platelet Rich Plasma). **Supplementary Fig. 31.** Forrest plot for the effect of PRP-therapy on live birth. (CI: Confidence Interval, PRP: Platelet Rich Plasma). **Supplementary Fig. 32.** Forrest plot for the effect of PRP-therapy on live birth after leaving Nazari 2022a. (CI: Confidence Interval, PRP: Platelet Rich Plasma). **Supplementary Fig. 34.** meta-regression for the effect of number of previous cycles on clinical pregnancy in women with implantation failure.

## Data Availability

All data generated or analyzed during this study are included in this published article or in the data repositories listed in References.
