# Supplementary material for: The efficacy of intrauterine infusion of platelet rich plasma in women undergoing assisted reproduction: a systematic review and meta-analysis
Source: BMC Pregnancy Childbirth. 2023 Dec 8;23:843. doi: 10.1186/s12884-023-06140-0 (PMC10704776; doi:10.1186/s12884-023-06140-0)
Supplement: Supplementary file 1 — Additional file 1: Supplementary Table 1. Quality assessment of the nonrandomized trials using ROBINS-I. Supplementary Table 2. Methodological quality assessment of the included 4 studies, based on the NOS for assessing the quality of epidemiological studies. Supplementary Fig. 1. Funnel plot of the meta-analysis of published studies for the clinical pregnancy rate. (RR: Relative Risk, SE: Standard Error). Supplementary Fig. 2. Funnel plot of the meta-analysis of published studies for the chemical pregnancy rate. (RR: Relative Risk, SE: Standard Error). Supplementary Fig. 3. Funnel plot of the meta-analysis of published studies for the miscarriage rate. (RR: Relative Risk, SE: Standard Error). Supplementary Fig. 4. Forrest plot for the effect of PRP-therapy on the live birth rate before leave one out. (CI: Confidence Interval, PRP: Platelet Rich Plasma). Supplementary Fig. 5. Forrest plot for the effect of PRP-therapy on endometrial thickness change. (CI: Confidence Interval, PRP: Platelet Rich Plasma). Supplementary Fig. 6. Forrest plot for the effect of PRP-therapy on implantation rate before leave one out. (CI: Confidence Interval, PRP: Platelet Rich Plasma). Supplementary Fig. 7. Forrest plot for the effect of PRP-therapy on implantation rate after leave one out. (CI: Confidence Interval, PRP: Platelet Rich Plasma). Supplementary Fig. 8. Forrest plot for the effect of PRP-therapy on chemical pregnancy rate. (CI: Confidence Interval, PRP: Platelet Rich Plasma). Supplementary Fig. 9. Forrest plot for the effect of PRP-therapy on ongoing pregnancy rate. (CI: Confidence Interval, PRP: Platelet Rich Plasma). Supplementary Fig. 10. Forrest plot for the effect of PRP-therapy on ectopic pregnancy rate. (CI: Confidence Interval, PRP: Platelet Rich Plasma). Supplementary Fig. 11. Forrest plot for the effect of PRP-therapy on multiple pregnancy rate. (CI: Confidence Interval, PRP: Platelet Rich Plasma). Supplementary Fig. 12. Forrest plot for the effect of PRP-therapy on c [file 12884_2023_6140_MOESM1_ESM.docx]

**The efficacy of intrauterine infusion of platelet rich plasma in women undergoing assisted reproduction: A systematic review and meta-analysis.**

**Search strategy:-**

**PubMed:**

("Platelet-Rich Plasma"[Mesh] OR Platelet-Rich Plasma OR PRP OR Platelet Rich Plasma OR Autologous platelet‐rich plasma OR Autologous platelet Rich plasma OR Autologous Conditioned plasma OR Platelet-rich plasma Gel OR Platelet Rich plasma Gel OR platelet Gel OR PRP Gel) AND ("Fertilization in Vitro"[Mesh] OR In Vitro Fertilization OR IVF OR In Vitro Fertilizations OR Test-Tube Fertilization OR Test-Tube Fertilizations OR Test Tube Fertilization OR Fertilizations in Vitro OR Test-Tube Babies OR Test Tube Babies OR Test-Tube Baby OR Recurrent in Vitro Fertilization Failure OR Recurrent in-vitro Fertilization Failure OR Recurrent IVF failure OR Recurrent Failure of in vitro Fertilization OR "Sperm Injections, Intracytoplasmic"[Mesh] OR Intracytoplasmic Sperm Injection OR Intracytoplasmic Sperm Injections OR ICSI OR "Embryo Transfer"[Mesh] OR Embryo Transfer OR Embryo Transfers OR Blastocyst Transfer OR Tubal Embryo Transfer OR Tubal Embryo Stage Transfer OR Embryo Implantation OR Poor Implantation OR Implantation Failure OR Repeated Implantation Failure OR Recurrent Implantation Failure OR Recurrent Failure of Implantation OR Recurrent Failure to Implant OR Repeat Failure To Implant OR Recurrent Failed Implantation OR Repeat Failed Implantation OR RIF OR Failed Cycle OR Recurrent Reproductive Failure OR Repeat Reproductive Failure OR "Reproductive Techniques"[Mesh] OR Reproductive Technique OR Reproductive Techniques OR Reproduction Technique OR Reproduction Techniques OR Reproduction Technic OR Reproduction Technics OR Reproductive Technology OR Reproductive Technologies OR "Reproductive Techniques, Assisted"[Mesh] OR Assisted Reproductive Technique OR Assisted Reproductive Techniques OR Assisted Reproductive Technic OR Assisted Reproductive Technics OR Assisted Reproductive Technology OR Assisted Reproductive Technologies)

Results: 486

Search fields: All fields

**Scopus:**

TITLE-ABS-KEY(“Platelet-Rich Plasma” OR “PRP” OR “Platelet Rich Plasma” OR “Autologous platelet‐rich plasma” OR “Autologous platelet Rich plasma” OR “Autologous Conditioned plasma” OR “Platelet-rich plasma Gel” OR “Platelet Rich plasma Gel” OR “platelet Gel” OR “PRP Gel”) AND (“In Vitro Fertilization” OR “IVF” OR “In Vitro Fertilizations” OR “Test-Tube Fertilization” OR “Test-Tube Fertilizations” OR “Test Tube Fertilization” OR “Fertilizations in Vitro” OR “Test-Tube Babies” OR “Test Tube Babies” OR “Test-Tube Baby” OR “Recurrent in Vitro Fertilization Failure” OR “Recurrent in-vitro Fertilization Failure” OR “Recurrent IVF failure” OR “Recurrent Failure of in vitro Fertilization” OR “Intracytoplasmic Sperm Injection” OR “Intracytoplasmic Sperm Injections” OR ICSI OR “Embryo Transfer” OR “Embryo Transfers” OR “Blastocyst Transfer” OR “Tubal Embryo Transfer” OR “Tubal Embryo Stage Transfer” OR “Embryo Implantation” OR “Poor Implantation” OR “Implantation Failure” OR “Repeated Implantation Failure” OR “Recurrent Implantation Failure” OR “Recurrent Failure of Implantation” OR “Recurrent Failure to Implant” OR “Repeat Failure To Implant” OR “Recurrent Failed Implantation” OR “Repeat Failed Implantation” OR “RIF” OR “Failed Cycle” OR “Recurrent Reproductive Failure” OR “Repeat Reproductive Failure” OR “Reproductive Technique” OR “Reproductive Techniques” OR “Reproduction Technique” OR “Reproduction Techniques” OR “Reproduction Technic” OR “Reproduction Technics” OR “Reproductive Technology” OR “Reproductive Technologies” OR “Assisted Reproductive Technique” OR “Assisted Reproductive Techniques” OR “Assisted Reproductive Technic” OR “Assisted Reproductive Technics” OR “Assisted Reproductive Technology” OR “Assisted Reproductive Technologies”)

Results: 201

**Web of Science:**

TS=(Platelet-Rich Plasma OR PRP OR Platelet Rich Plasma OR Autologous platelet‐rich plasma OR Autologous platelet Rich plasma OR Autologous Conditioned plasma OR Platelet-rich plasma Gel OR Platelet Rich plasma Gel OR platelet Gel OR PRP Gel) AND TS= (In Vitro Fertilization OR IVF OR In Vitro Fertilizations OR Test-Tube Fertilization OR Test-Tube Fertilizations OR Test Tube Fertilization OR Fertilizations in Vitro OR Test-Tube Babies OR Test Tube Babies OR Test-Tube Baby OR Recurrent in Vitro Fertilization Failure OR Recurrent in-vitro Fertilization Failure OR Recurrent IVF failure OR Recurrent Failure of in vitro Fertilization OR Intracytoplasmic Sperm Injection OR Intracytoplasmic Sperm Injections OR ICSI OR Embryo Transfer OR Embryo Transfers OR Blastocyst Transfer OR Tubal Embryo Transfer OR Tubal Embryo Stage Transfer OR Embryo Implantation OR Poor Implantation OR Implantation Failure OR Repeated Implantation Failure OR Recurrent Implantation Failure OR Recurrent Failure of Implantation OR Recurrent Failure to Implant OR Repeat Failure To Implant OR Recurrent Failed Implantation OR Repeat Failed Implantation OR RIF OR Failed Cycle OR Recurrent Reproductive Failure OR Repeat Reproductive Failure OR Reproductive Technique OR Reproductive Techniques OR Reproduction Technique OR Reproduction Techniques OR Reproduction Technic OR Reproduction Technics OR Reproductive Technology OR Reproductive Technologies OR Assisted Reproductive Technique OR Assisted Reproductive Techniques OR Assisted Reproductive Technic OR Assisted Reproductive Technics OR Assisted Reproductive Technology OR Assisted Reproductive Technologies)

Results: 366

**Cochrane:**

1. Platelet-Rich Plasma OR PRP OR Platelet Rich Plasma OR Autologous platelet‐rich plasma OR Autologous platelet Rich plasma OR Autologous Conditioned plasma OR Platelet-rich plasma Gel OR Platelet Rich plasma Gel OR platelet Gel OR PRP Gel
2. In Vitro Fertilization OR IVF OR In Vitro Fertilizations OR Test-Tube Fertilization OR Test-Tube Fertilizations OR Test Tube Fertilization OR Fertilizations in Vitro OR Test-Tube Babies OR Test Tube Babies OR Test-Tube Baby OR Recurrent in Vitro Fertilization Failure OR Recurrent in-vitro Fertilization Failure OR Recurrent IVF failure OR Recurrent Failure of in vitro Fertilization OR Intracytoplasmic Sperm Injection OR Intracytoplasmic Sperm Injections OR ICSI OR Embryo Transfer OR Embryo Transfers OR Blastocyst Transfer OR Tubal Embryo Transfer OR Tubal Embryo Stage Transfer OR Embryo Implantation OR Poor Implantation OR Implantation Failure OR Repeated Implantation Failure OR Recurrent Implantation Failure OR Recurrent Failure of Implantation OR Recurrent Failure to Implant OR Repeat Failure To Implant OR Recurrent Failed Implantation OR Repeat Failed Implantation OR RIF OR Failed Cycle OR Recurrent Reproductive Failure OR Repeat Reproductive Failure OR Reproductive Technique OR Reproductive Techniques OR Reproduction Technique OR Reproduction Techniques OR Reproduction Technic OR Reproduction Technics OR Reproductive Technology OR Reproductive Technologies OR Assisted Reproductive Technique OR Assisted Reproductive Techniques OR Assisted Reproductive Technic OR Assisted Reproductive Technics OR Assisted Reproductive Technology OR Assisted Reproductive Technologies
3. #1 AND #2

Results: 153

**Supplementary Table 1.** Quality assessment of the nonrandomized trials using ROBINS-I

| **Study**  **Domain** | **Abou-El-Naga et al 2022** | **Dzhincharadze et al 2021** | **Tehraninejad et al 2021** |
| --- | --- | --- | --- |
| **Pre-intervention** |  |  |  |
| Bias due to confounding | Low risk | Low risk of bias | Low risk of bias |
| Bias in selection of participants into the study | Low risk | Low risk of bias | Low risk of bias |
| **At intervention** |  |  |  |
| Bias in classification of interventions | Low risk | Low risk of bias | Low risk |
| **Post-intervention** |  |  |  |
| Bias due to deviations from intended interventions | Low risk | Low risk of bias | Low risk |
| Bias due to missing data | Low risk | Low risk | Low risk |
| Bias in measurement of outcomes | Low risk | Low risk of bias | Low risk |
| Bias in selection of the reported result | Low risk | Moderate risk of bias | Moderate risk of bias |
| **Overall risk of bias** | Low risk | Moderate risk of bias | Moderate risk of bias |

**S*upplementary Table 2***. Methodological quality assessment of the included 4 studies, based on the NOS for assessing the quality of epidemiological studies.

Cohort Studies (n=6)

| Study | Selection |  |  |  | Comparability | Outcome |  | |  | Total Score |
| --- | --- | --- | --- | --- | --- | --- | --- | --- | --- | --- |
|  | Representativeness of the exposed cohort | Selection of the non-exposed cohort | Ascertainment of exposure5 | Outcome was not present at start of study6 | Control for 2 important factors2,3 | Assessment of outcome | Follow-up long enough | Adequacy of follow-up of cohort7 | |  |
| chang et al 2019 |  | * | * | * | ** | * |  | | * | 7 |
| Coksuer et al 2019 |  | * | * | * | ** | * | * | | * | 8 |
| Noushin et al 2021 |  | * | * | * | ** | * | * | | * | 8 |
| Xu et al 2022 |  | * | * | * | ** | * | * | | * | 8 |
| yuan et al 2022 |  | * |  | * | ** | * | * | | * | 7 |
| ban et al 2023 |  | * |  | * | ** | * |  | | * | 6 |


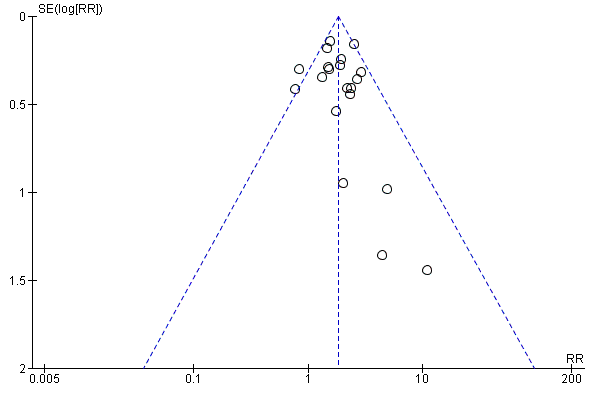


Supplementary Fig.1. Funnel plot of the meta-analysis of published studies for the clinical pregnancy rate. (RR: Relative Risk, SE: Standard Error).


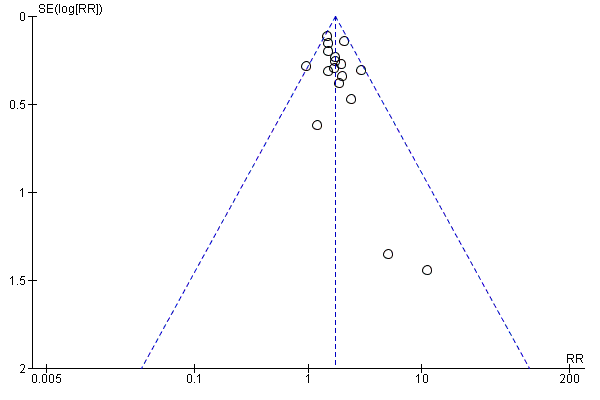


Supplementary Fig. 2. Funnel plot of the meta-analysis of published studies for the chemical pregnancy rate. (RR: Relative Risk, SE: Standard Error).


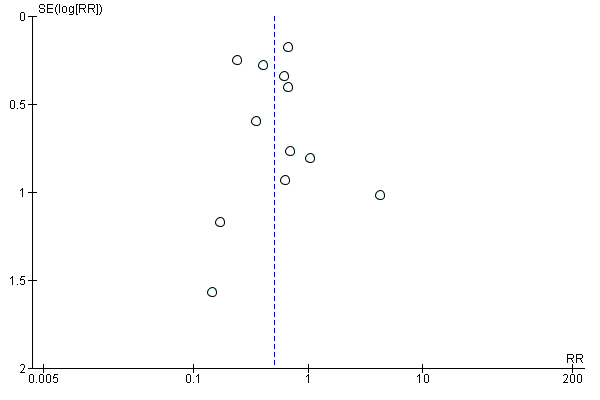


Supplementary Fig. 3. Funnel plot of the meta-analysis of published studies for the miscarriage rate. (RR: Relative Risk, SE: Standard Error).


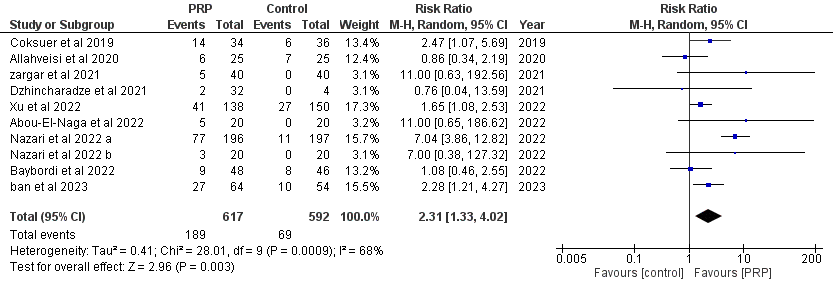


Supplementary Fig. 4. Forrest plot for the effect of PRP-therapy on the live birth rate before leave one out. (CI: Confidence Interval, PRP: Platelet Rich Plasma).


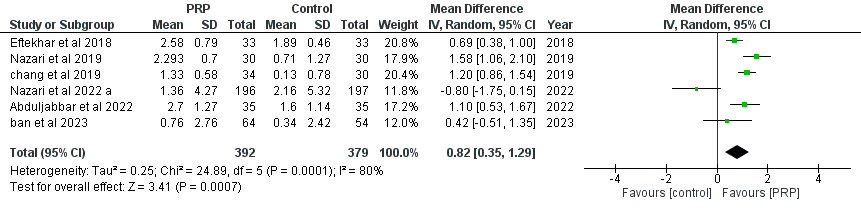


Supplementary Fig. 5. Forrest plot for the effect of PRP-therapy on endometrial thickness change. (CI: Confidence Interval, PRP: Platelet Rich Plasma).


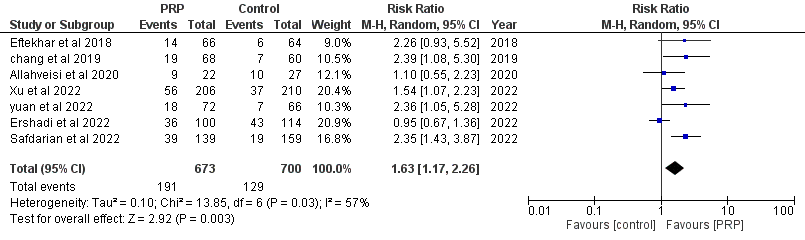


Supplementary Fig. 6 .Forrest plot for the effect of PRP-therapy on implantation rate before leave one out. (CI: Confidence Interval, PRP: Platelet Rich Plasma).


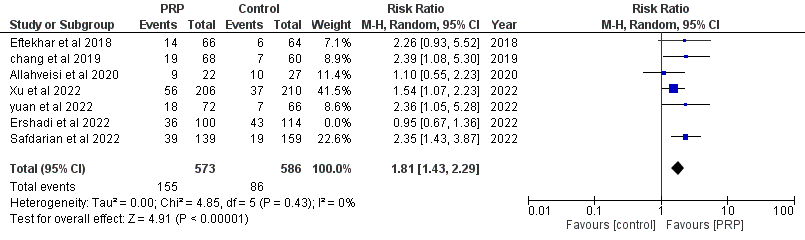


Supplementary Fig.7. Forrest plot for the effect of PRP-therapy on implantation rate after leave one out. (CI: Confidence Interval, PRP: Platelet Rich Plasma).


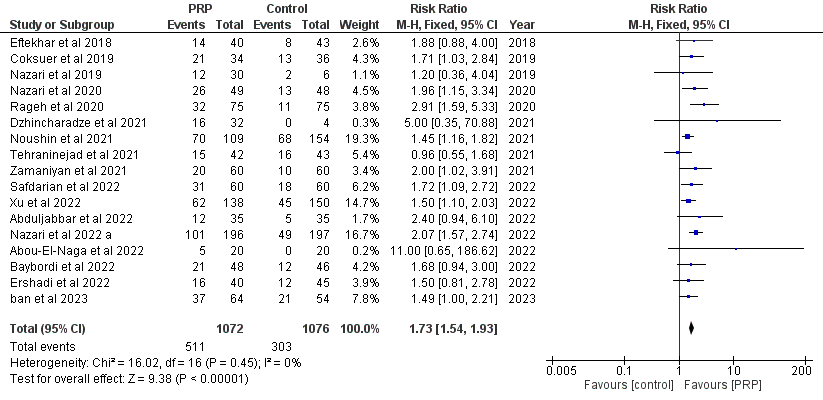


Supplementary Fig.8. Forrest plot for the effect of PRP-therapy on chemical pregnancy rate. (CI: Confidence Interval, PRP: Platelet Rich Plasma).


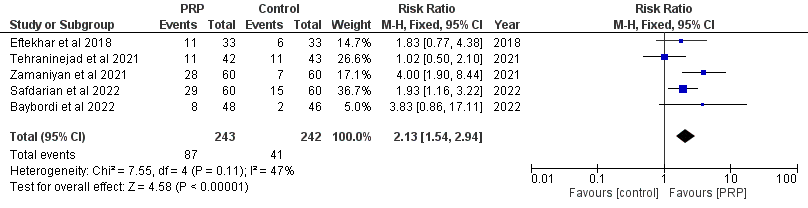


Supplementary Fig.9. Forrest plot for the effect of PRP-therapy on ongoing pregnancy rate. (CI: Confidence Interval, PRP: Platelet Rich Plasma).


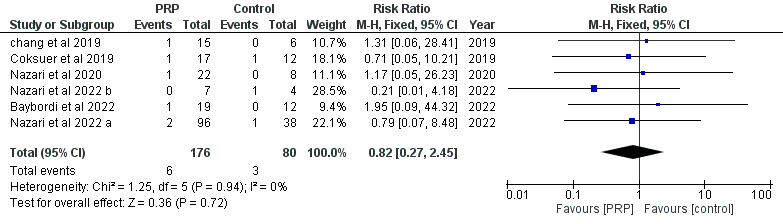


Supplementary Fig.10. Forrest plot for the effect of PRP-therapy on ectopic pregnancy rate. (CI: Confidence Interval, PRP: Platelet Rich Plasma).

**
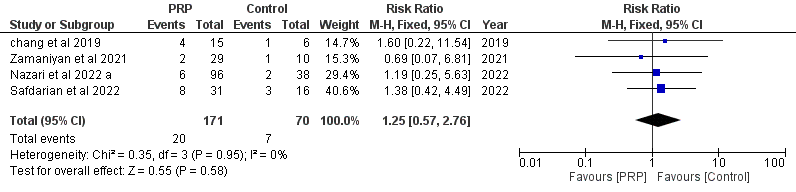
**

Supplementary Fig.11. Forrest plot for the effect of PRP-therapy on multiple pregnancy rate. (CI: Confidence Interval, PRP: Platelet Rich Plasma).

---------------------------------------------

RCTs


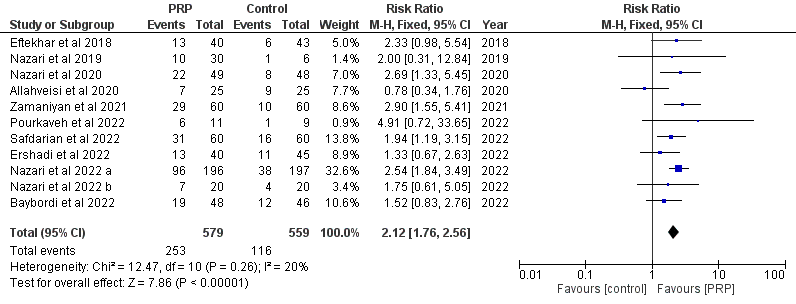


Supplementary Fig.12. Forrest plot for the effect of PRP-therapy on clinical pregnancy rate in RCT studies. (CI: Confidence Interval, PRP: Platelet Rich Plasma).


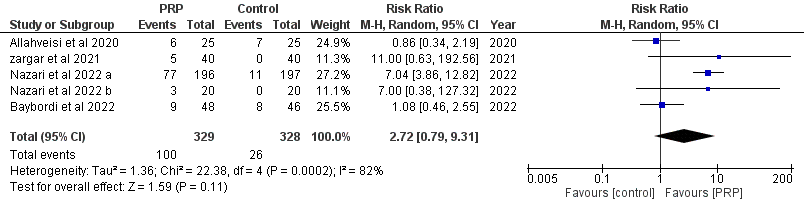


Supplementary Fig.13. Forrest plot for the effect of PRP-therapy on the live birth rate before leave one out in RCT studies. (CI: Confidence Interval, PRP: Platelet Rich Plasma).


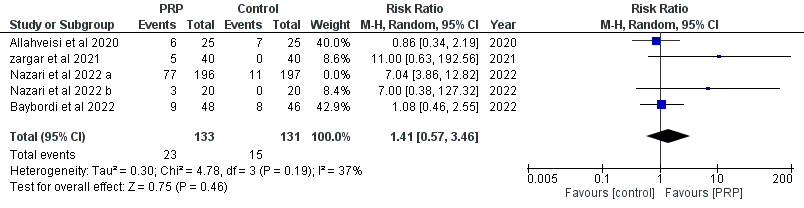


Supplementary Fig.14. Forrest plot for the effect of PRP-therapy on live birth rate after leave one out in RCT studies. (CI: Confidence Interval, PRP: Platelet Rich Plasma).


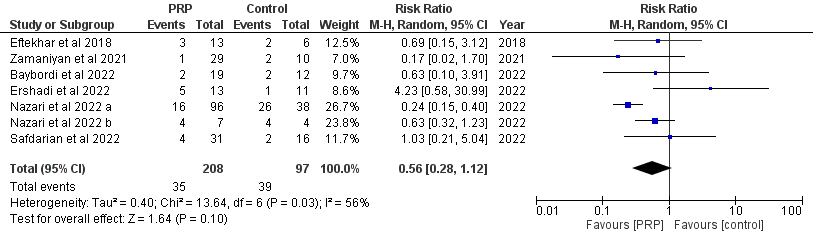


Supplementary Fig.15. Forrest plot for the effect of PRP-therapy on miscarriage rate before leave one out in RCT studies. (CI: Confidence Interval, PRP: Platelet Rich Plasma).


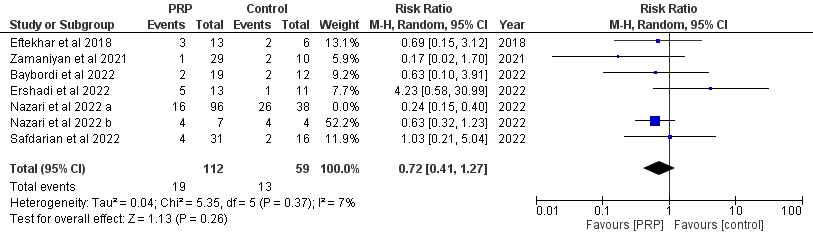


Supplementary Fig.16. Forrest plot for the effect of PRP-therapy on miscarriage rate after leave one out in RCT studies. (CI: Confidence Interval, PRP: Platelet Rich Plasma).

-------------------------------------------------------------------------------

RIF


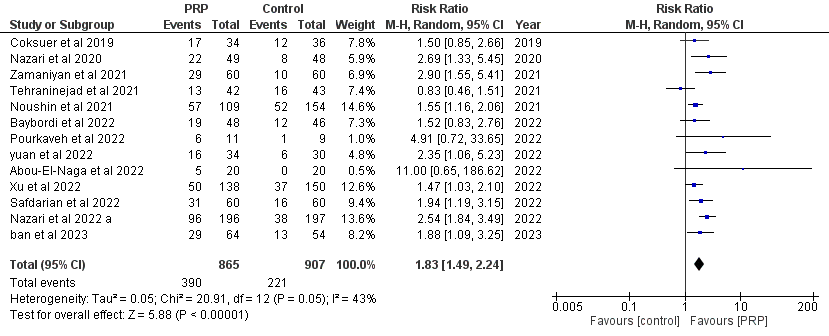


Supplementary Fig.17. Forrest plot for the effect of PRP-therapy on clinical pregnancy rate in patients with RIF (CI: Confidence Interval, PRP: Platelet Rich Plasma).


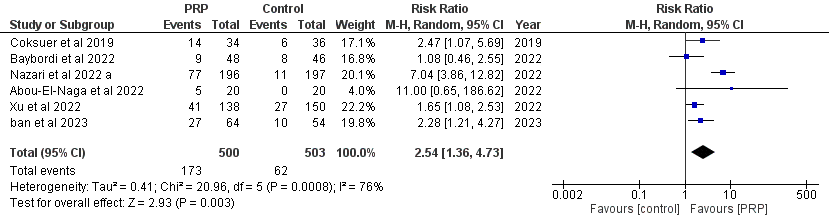


Supplementary Fig.18 Forrest plot for the effect of PRP-therapy on the live birth rate before leave one out in patients with RIF. (CI: Confidence Interval, PRP: Platelet Rich Plasma)


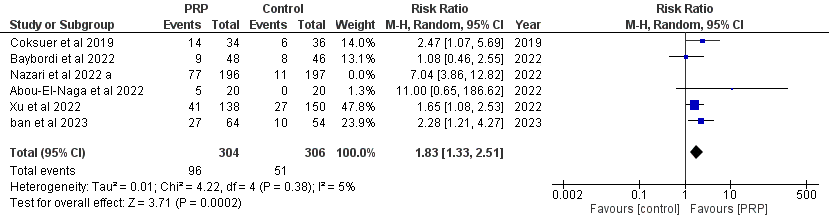


Supplementary Fig.19 Forrest plot for the effect of PRP-therapy on the live birth rate after leave one out in patients with RIF. (CI: Confidence Interval, PRP: Platelet Rich Plasma)


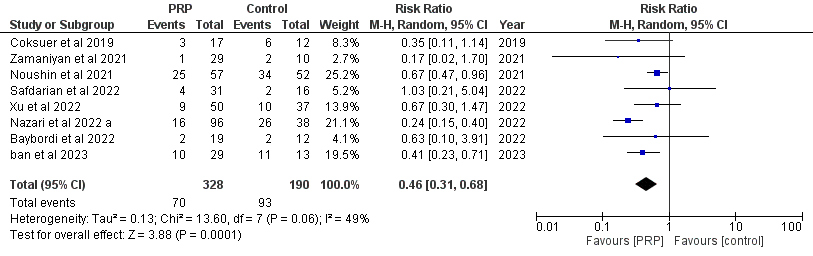


Supplementary Fig.20. Forrest plot for the effect of PRP-therapy on miscarriage rate in patients with RIF. (CI: Confidence Interval, PRP: Platelet Rich Plasma).

-----------------------------------------------------------------------

Thin endometrium


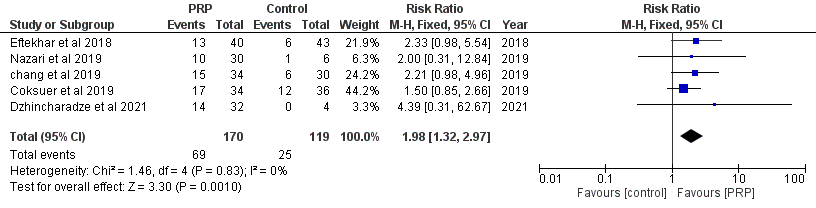


Supplementary Fig.21. Forrest plot for the effect of PRP-therapy on clinical pregnancy rate one in patients with thin endometrium. (CI: Confidence Interval, PRP: Platelet Rich Plasma).


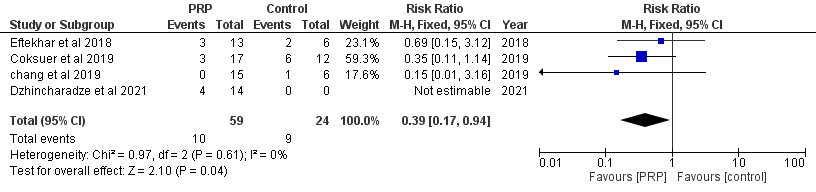


Supplementary Fig.22. Forrest plot for the effect of PRP-therapy on miscarriage rate in patients with thin endometrium. (CI: Confidence Interval, PRP: Platelet Rich Plasma).

--------------------------------------------

Sub group based on dose and age


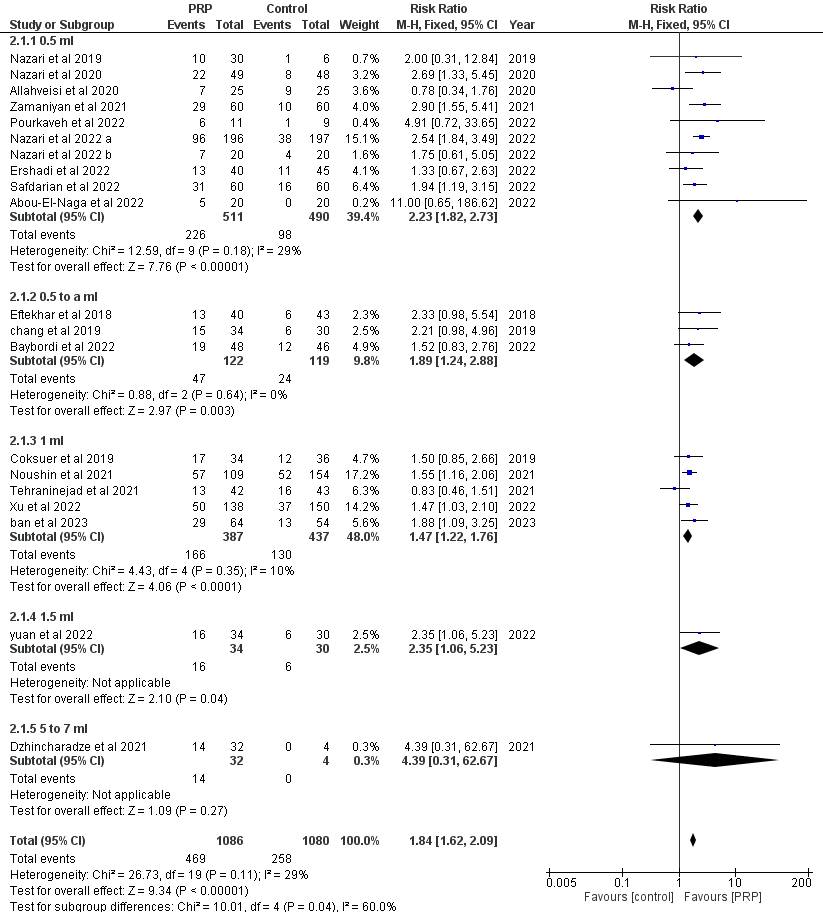


Supplementary Fig.23. Forrest plot for the effect of PRP-therapy on clinical pregnancy rate. (CI: Confidence Interval, PRP: Platelet Rich Plasma).


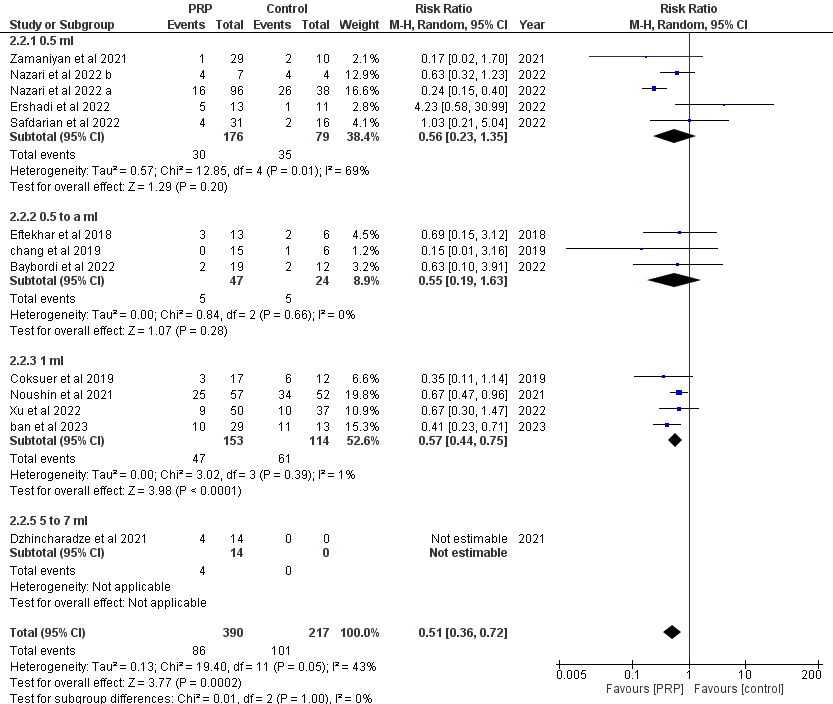


Supplementary Fig.24. Forrest plot for the effect of PRP-therapy on miscarriages. (CI: Confidence Interval, PRP: Platelet Rich Plasma).


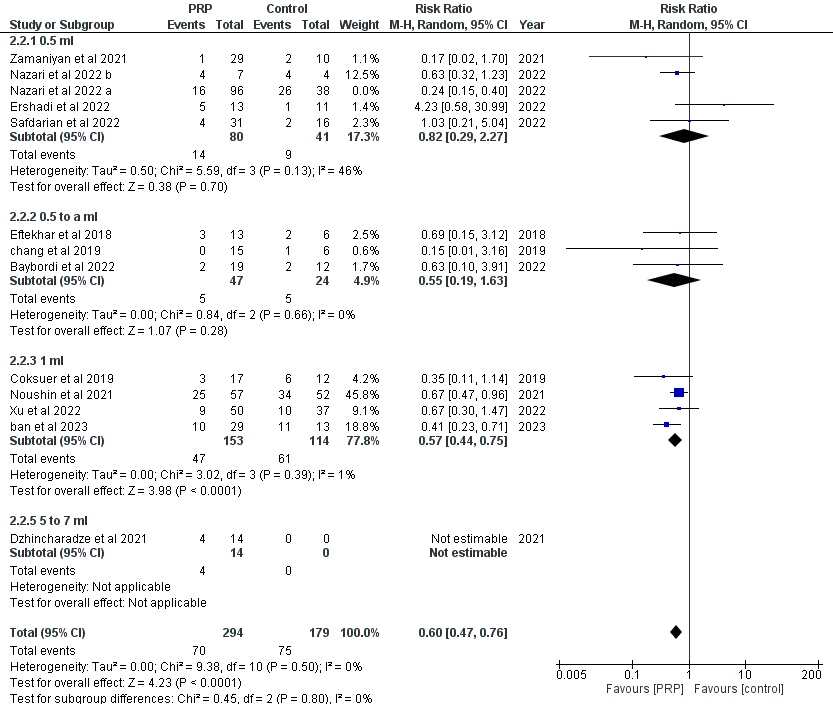


Supplementary Fig.25. Forrest plot for the effect of PRP-therapy on miscarriages after leaving Nazari 2022a. (CI: Confidence Interval, PRP: Platelet Rich Plasma).


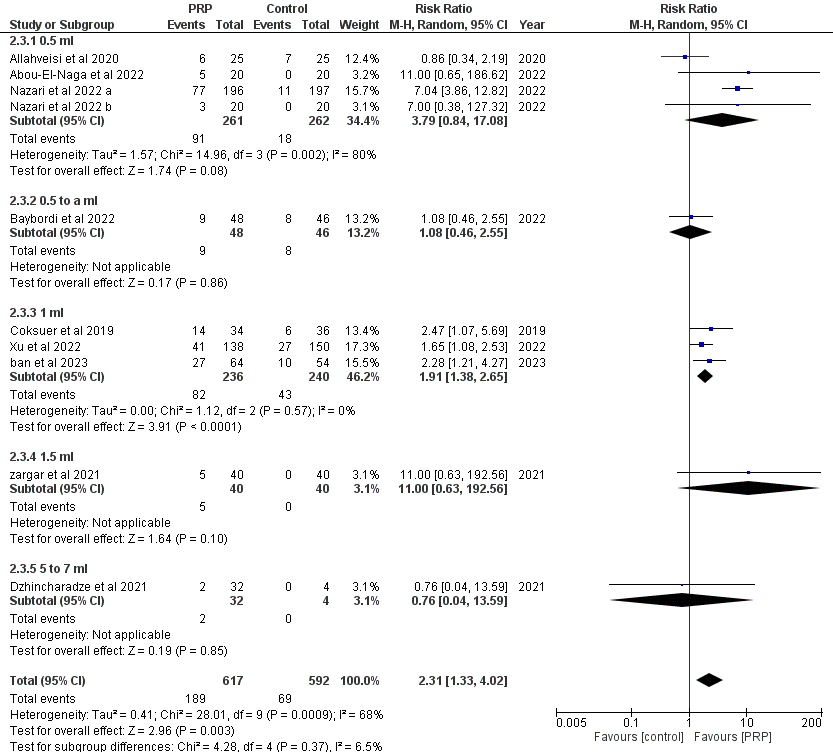


Supplementary Fig.26. Forrest plot for the effect of PRP-therapy on live birth. (CI: Confidence Interval, PRP: Platelet Rich Plasma).


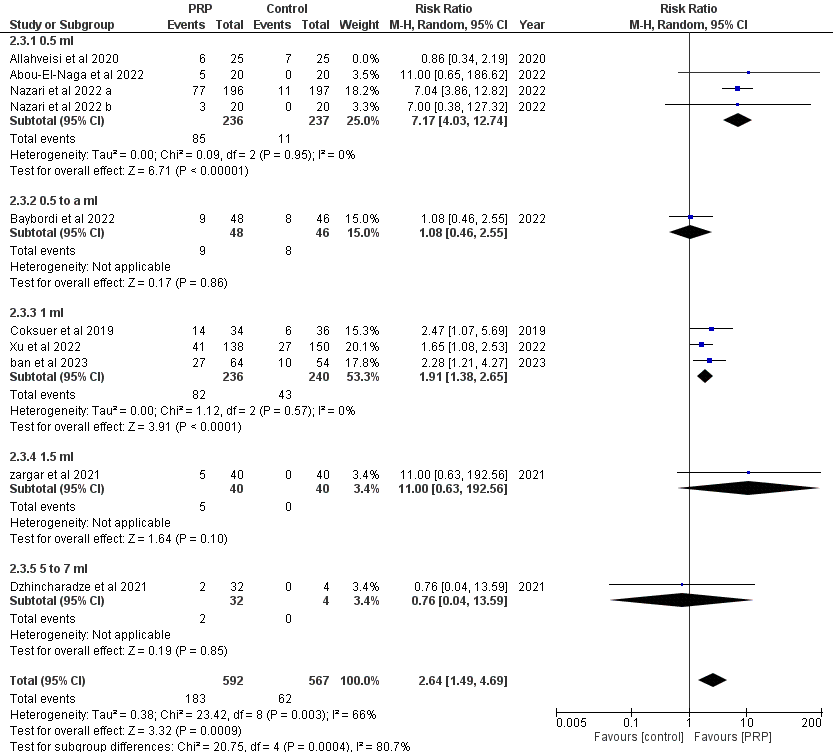


Supplementary Fig.27. Forrest plot for the effect of PRP-therapy on live birth after allahveisi. (CI: Confidence Interval, PRP: Platelet Rich Plasma).

**
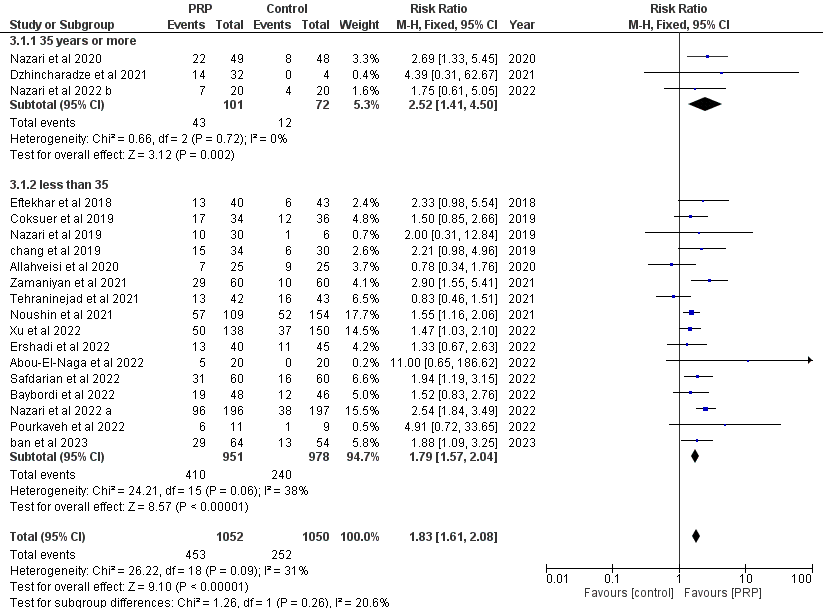
**

Supplementary Fig.28. Forrest plot for the effect of PRP-therapy on clinical pregnancy. (CI: Confidence Interval, PRP: Platelet Rich Plasma).


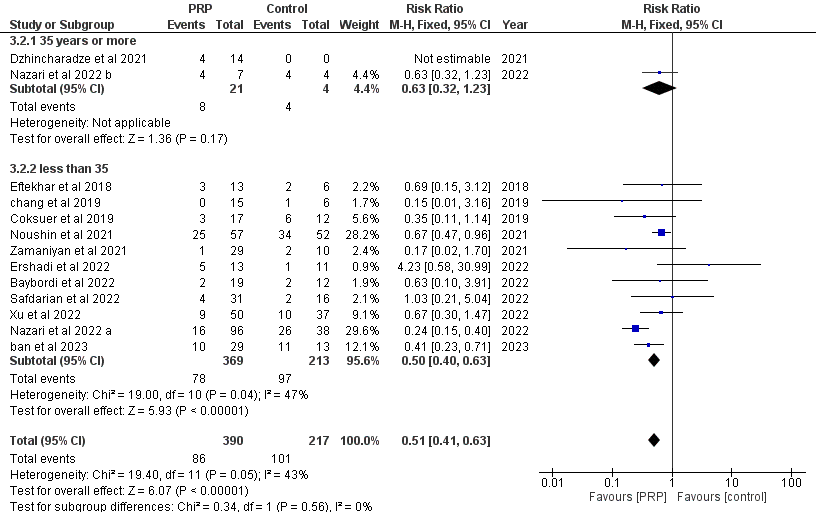


Supplementary Fig.29. Forrest plot for the effect of PRP-therapy on miscarriage. (CI: Confidence Interval, PRP: Platelet Rich Plasma).


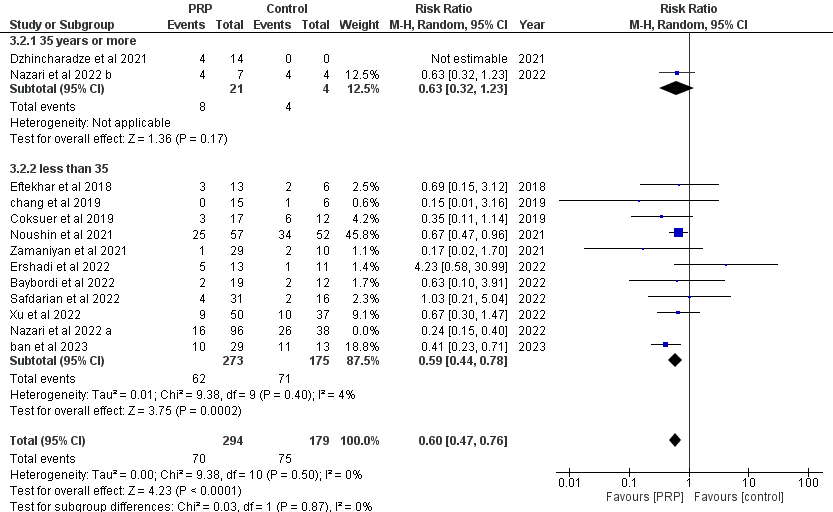


Supplementary Fig.30. Forrest plot for the effect of PRP-therapy on miscarriage after leaving Nazari 2022a. (CI: Confidence Interval, PRP: Platelet Rich Plasma).

**
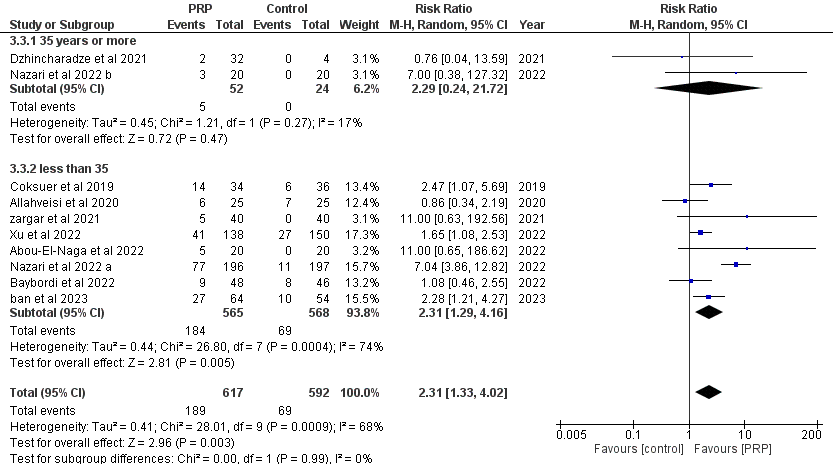
**

Supplementary Fig.31. Forrest plot for the effect of PRP-therapy on live birth. (CI: Confidence Interval, PRP: Platelet Rich Plasma).

**
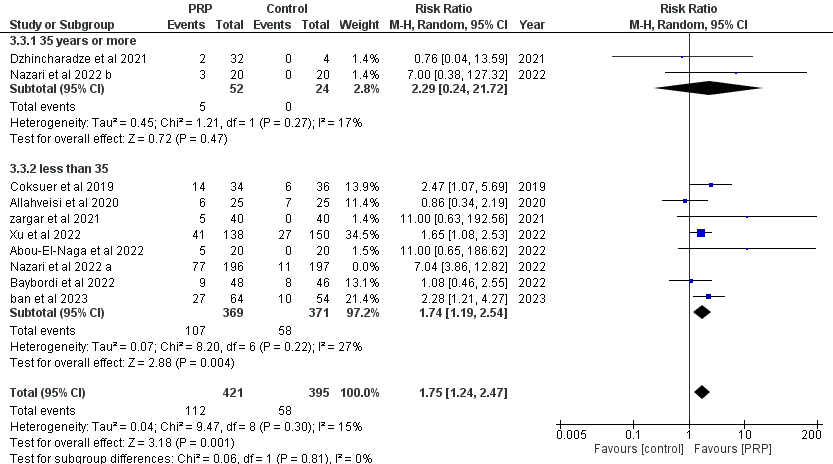
**

Supplementary Fig.32. Forrest plot for the effect of PRP-therapy on live birth after leaving Nazari 2022a. (CI: Confidence Interval, PRP: Platelet Rich Plasma).

--------------------------

Meta-regression


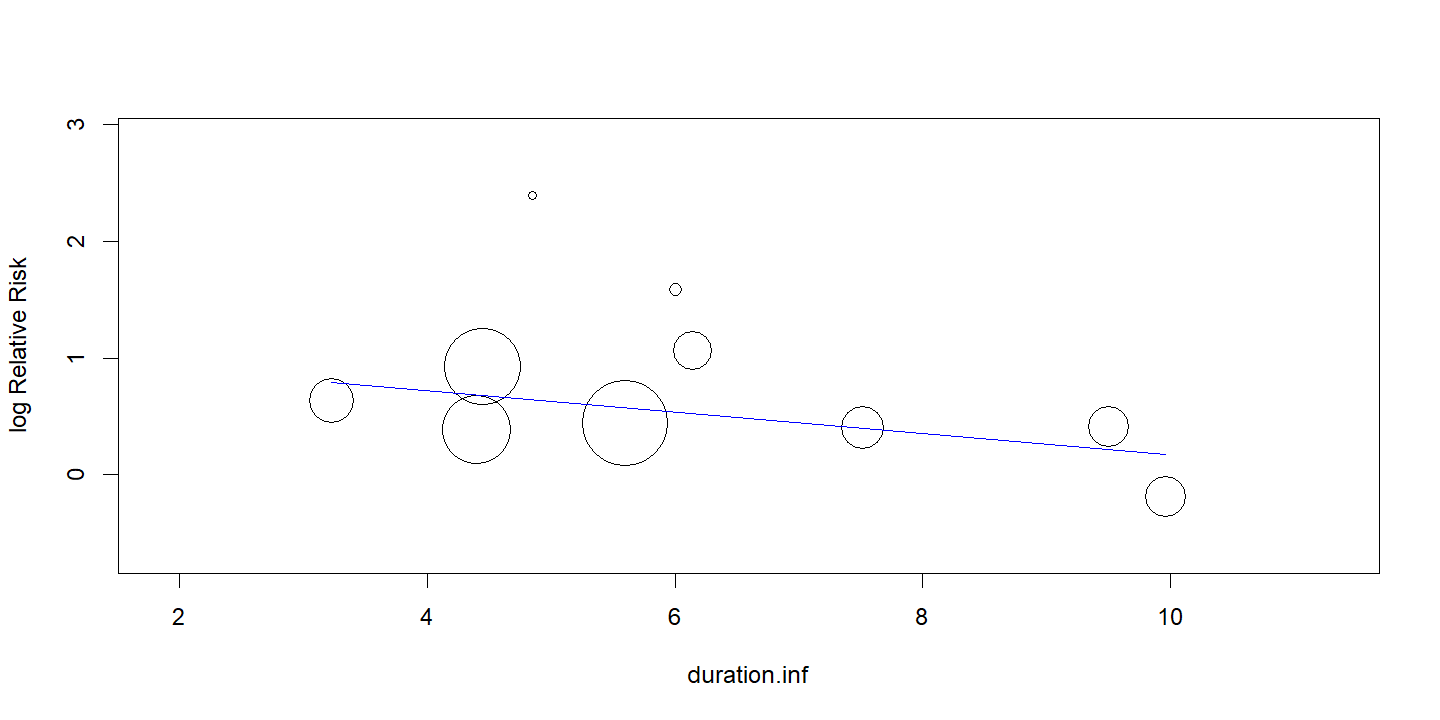


Supplementary Fig.33 meta-regression for the effect of duration of infertility on clinical pregnancy in women with RIF


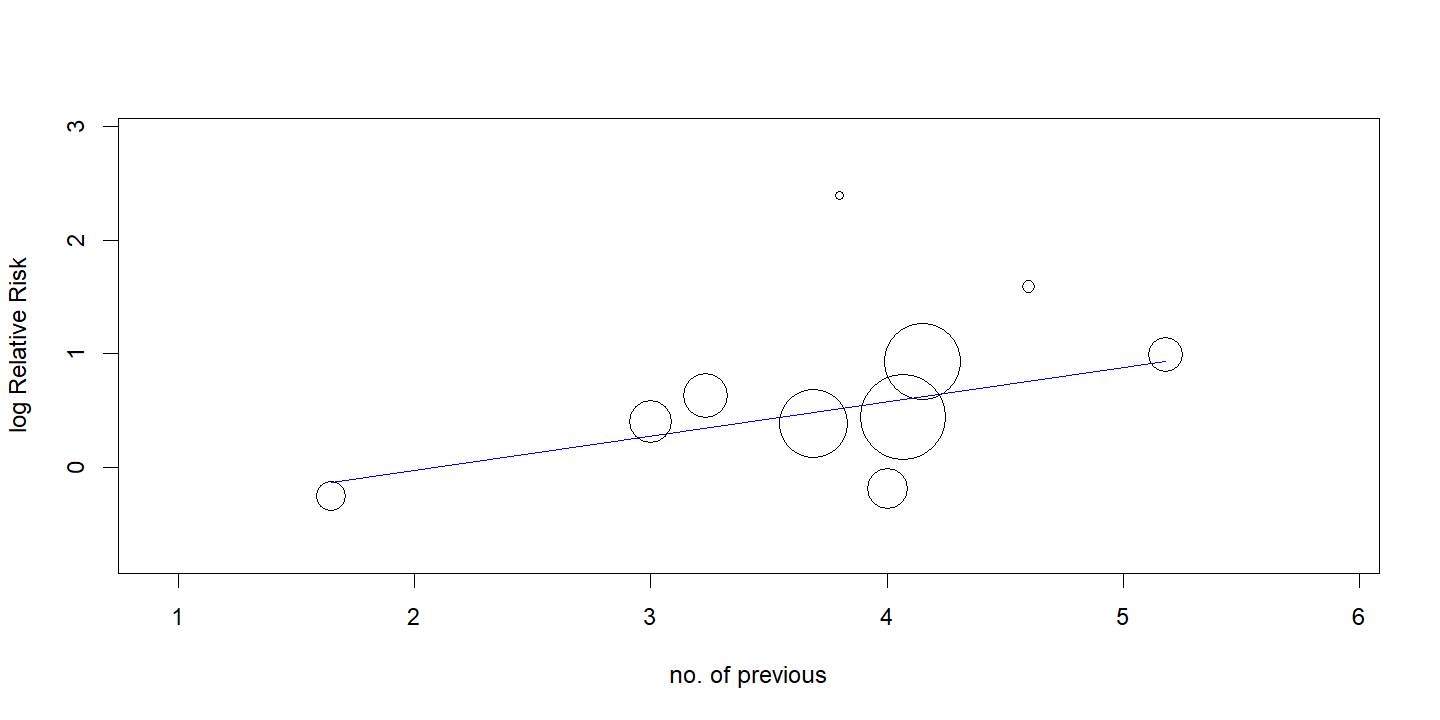


Supplementary Fig.34 meta-regression for the effect of number of previous cycles on clinical pregnancy in women with implantation failure
